# Research on multi-stage on-board detection algorithm of track defects of high-speed railway based on the influence mechanism of track defects

**DOI:** 10.1038/s41598-025-34483-5

**Published:** 2026-01-02

**Authors:** Jianbo Li, Hongmei Shi, Ji Qiu, Jiaqi Shi, Zujun Yu, Zengqiang Jiang

**Affiliations:** 1 State Key Laboratory of Advanced Rail Autonomous Operation, Beijing Jiaotong University, Beijing, China; 2 School of Mechanical, Electronic and Control Engineering, Beijing Jiaotong University, Beijing, China

**Keywords:** Vehicle-track coupling model, Track defects, On-board detection, Intelligent algorithm, Engineering, Civil engineering, Mechanical engineering

## Abstract

The ballastless track defects, such as rail corrugation, fastener looseness, and CA mortar layer disengagement, will affect the safety of high-speed train operation. Hence, a multi-stage on-board detection algorithm of track defects based on the influence mechanism of track defects is proposed for track condition monitoring. Firstly, based on refined and discrete track models, a multi-scale track defect model and a segmental numerical solution method are developed, which greatly improve the solving efficiency. Then, based on the multi-scale vehicle-track coupling model with track defects, the effect of track defects on wheelset vibration response is analysed from multiple dimensions. Simultaneously, a track defect characteristic decoupling algorithm based on signal decomposition and time-frequency analysis is suggested, and a series of characteristic indexes representing track defects are constructed. Finally, a multi-stage intelligent recognition algorithm of track defects based on the nested combination of machine learning, deep learning, and transfer learning is proposed‌. In the first stage, the identification of track defect types is achieved using wheelset vibration feature vectors and the nonlinear classifier model. In the second stage, a multi-channel track defect degree recognition model is designed based on a deep residual network and multiple wheelset data, achieving accurate estimation of rail corrugation depth, fastener looseness number, and CA mortar disengagement length. In the third stage, a transfer learning network for the track defect degree recognition model based on multiple moment matching is put forward, which enables the transfer of the track defect detection algorithm for different vehicles.

## Introduction

Ballastless track is widely used in the Chinese high-speed railway. The primary defects in ballastless track include rail corrugation, fastener looseness, and cement asphalt mortar (CA mortar) layer disengagement. The most common detection techniques for track defects include machine vision, inertial measurement, ground-penetrating radar, and methods based on vehicle vibration response. The track defect detection method based on dynamic response signals of operating vehicles is cost-effective, offers frequent monitoring, and does not interfere with the regular operation of high-speed, high-density railways.

The onboard detection method, based on vehicle vibration response, is commonly used to assess track irregularity and monitor the track health status. Liu et al.^1^ employed the fractal analysis method to investigate the relationship between track geometric irregularity and the vertical and lateral accelerations of the car body. Xiao et al.^2^ utilized sigma point sets to simulate random parameters and noise, and developed a vehicle track irregularity recognition algorithm based on Bayesian Kalman filter technology. In addition to the car body of the vehicle, the bogie vibration response can also reflect the condition of the track. Lathe et al.^3^ employed the extended Kalman particle filter method to analyze the signals of vertical acceleration and pitch angle of the bogie, yielding an estimate of the vertical irregularity. Guo et al.^4^ deployed multiple sensors on the vehicle and bogie, and proposed a track irregularity estimation method based on the measurement system attitude and an unknown input observer estimator. The wheelset directly contacts the rail of the track, so more track state detection algorithms rely on axle box acceleration. Sun et al.^5^ created a longitudinal irregularity measurement algorithm based on axle box acceleration, utilizing the inertial reference method and multiple digital filters. Agh et al.^6^ found a strong correlation between the second-order spatial derivative of lateral track irregularity and the lateral acceleration of the axle box.

Based on the evaluation of track irregularity, the on-board inspection method can also identify surface defects on the rail. Ng et al.^7^ analyzed the acceleration signal of axle box vibration using short-time Fourier transform, continuous wavelet transform, empirical mode decomposition, and power spectral density, and examined the relationship between axle box acceleration and the severity of rail defects. Yan et al.^8^ employed an improved synchronous compression wavelet transform to extract the instantaneous frequency of high-speed comprehensive detection train axle box acceleration data, thereby identifying and locating rail corrugation and grinding marks. Haji Abdulrazagh et al.^9^ utilized axle box vertical vibration acceleration to measure the surface roughness of the rail, and compared the roughness calculation results with those obtained from the track inspection car to verify the reliability and accuracy of the method. Shadfar et al.^10^ employed the Fourier transform, empirical mode decomposition, and ensemble empirical mode decomposition methods to analyse the axle box acceleration of subway trains and detect rail defects. Hoelzl et al.^11^ established an expert evaluation database, and fused the characteristics of axle box vibration acceleration data with expert feedback information to evaluate the defects of rail welds. Liu et al.^12^ collected the axle box vibration response and proposed a rail corrugation identification method based on empirical mode decomposition, entropy, and Wigner-Ville distribution. Naseri et al.^13^ used a hybrid method combining wavelet packet analysis and Hilbert-Huang transform to detect spot irregularities.

Besides detecting surface defects on rails and monitoring track irregularity, methods based on vehicle vibration response analysis are also essential for inspecting track structures and subsurface defects. Zhang et al.^14^ found that the natural frequency of the vehicle system varies periodically with the vehicle position. Based on changes in vehicle vibrations when fasteners are lost or ballast is damaged, they proposed a method for identifying rail support failure using the vertical acceleration of the detection vehicle. Likewise, the Hilbert-Huang transform energy entropy evaluation algorithm is used to analyze the vibration signal of the wheelset, and then the looseness of the fasteners can be identified by calculating the energy entropy of the key IMF components of the signal^15^. Malekjafarian et al.^16^ proposed a method for track monitoring using acceleration sensors installed on passenger cars. The technique employs numerical integration and bandpass filters to calculate the displacement of the bogie from its vertical acceleration, which is then used to identify track defects such as rail sleeper suspension. Auersch et al.^17^ found that when the stiffness of the rail support varies along the track, an additional low-frequency component is created by the excitation of the track on the vehicle. The vehicle vibration response can indicate changes in track stiffness, which can be used to detect structural defects in the track. Miao et al.^18^ conducted a high-order statistical analysis of the vertical axle box acceleration and proposed a method for locating and identifying the mortar voids in ballastless track. Vishwakarma et al.^19^ utilized a continuous wavelet transform of axle box vibration acceleration to detect the location and severity of defects in the track fastener.

With the development of machine learning and deep learning, some scholars have gradually carried out related theory and application research on intelligent detection algorithms for track defects. Chen et al.^20^ defined damage degrees by modifying the stiffness and damping coefficients of the fastener and designed a damage identification algorithm for the fastener based on a fully convolutional network, utilizing axle box acceleration to predict the damage degree of the fastener. Yang et al.^21^ employed a convolutional neural network and a multi-scale self-attention mechanism to predict track geometric irregularity. Ren et al.^22^ combined feature extraction, support vector machine, and a convolutional neural network to accurately detect the subgrade settlement disease of ballastless track. Wang et al.^23^ performed an empirical wavelet transform on axle box acceleration and used a convolutional neural network and long short-term memory networks to identify and classify rail corrugation. Liu et al.^24^ employed adaptive chirp mode decomposition and recurrence plot to convert axle box acceleration into a two-dimensional image, and proposed an intelligent recognition method for short-wave irregularity based on a convolutional neural network.

In recent years, the method of track state detection based on vehicle dynamic response has become a research hotspot in the field of track defect detection and track health monitoring. However, there are still the following issues with on-board detection methods for track defect detection: (1) The theoretical foundation for onboard detection of high-speed railway track defects is weak, and research on detailed track defect simulation models that consider multiple track defects is lacking; (2) Using only one signal analysis method makes it hard to accurately separate track defect characteristics from the combined vehicle vibration response signal; (3) The usual detection algorithms rely heavily on fault samples to evaluate the severity of track defects, and because vibration responses vary among different vehicles, they cannot be directly applied across various vehicle types.

The paper explores intelligent algorithms for detecting high-speed railway track defects through vehicle dynamic responses. First, a multi-scale vehicle-track coupling model based on refined and discrete track models is developed, and a segmental numerical solution method is introduced to provide theoretical frameworks and data sources for track defect detection algorithm research. Second, an in-depth analysis examines the effects of rail corrugation, fastener looseness, and CA mortar layer disengagement on wheelset dynamic responses, offering prior knowledge for the decoupling algorithm of track defect characteristics. Third, a nonlinear track defect feature decoupling algorithm based on signal decomposition and time-frequency analysis is proposed to extract track defect feature indexes. Finally, a multi-stage intelligent recognition algorithm for track defects, combining traditional machine learning, deep learning, and transfer learning, is presented, enabling rapid identification of track defect types, precise recognition of defect severity, and transferability of the algorithm across different vehicles.

## Multi-scale vehicle-track coupling model with track defects

The types of track defects in high-speed railways are complex and varied, which can threaten the safety of high-speed railway operations. In researching track defect detection algorithms, it is challenging to perform line experiments by introducing track defects in an actual railway. Accordingly, the vehicle-track coupling dynamic model, which considers the effects of track defects, serves as the theoretical basis for studying the on-board perception mechanism and detection algorithms of high-speed railway track defects. A multi-scale high-speed vehicle and slab ballastless track coupling model, which includes a multi-scale track defect model and segmental numerical solution method, has been developed. The model incorporates three typical defects: rail corrugation, fastener looseness, and CA mortar layer disengagement.

### High-speed vehicle and slab ballastless track model

The main structure of high-speed train vehicles includes a body, two bogie frames, and four wheelsets. According to the theory of vehicle system dynamics^25^, different types of high-speed vehicles can be modeled as a multi-rigid-body system with a two-stage suspension and 35 degrees of freedom, represented as:

1 $${M_V}{\ddot {X}_V}+{C_V}{\dot {X}_V}+{K_V}{X_V}={P_V}$$

where, ${X_V}$ is displacement vector of the vehicle model; ${M_V}$ is mass matrix of the vehicle model; ${C_V}$ is damping matrix of the vehicle model; ${K_V}$is stiffness matrix of the vehicle model; ${P_V}$ is load vector of the vehicle model.

**Fig. 1:**
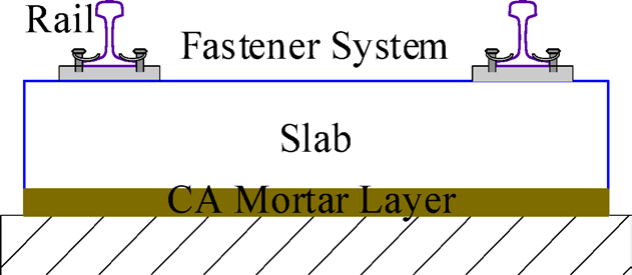
The model of high-speed railway slab ballastless track.

Most high-speed railways in China use ballastless track, with CRTS II slab ballastless tracks being widely implemented. The slab ballastless track system consists of the rail, fastener systems, track slabs, and the CA mortar layer, as illustrated in Fig. 1. The track model does not account for differences among various types of slab ballastless tracks and ignores the base plate or bridge structure. Therefore, the established slab ballastless track model is universal. Based on the Euler-Bernoulli beam equation and elastic thin plate theory^26^, the vibration equations of rail and slab can be derived. Using the Ritz method, the vibration equations can be converted into second-order ordinary differential equations that are solvable. Furthermore, the slab ballastless track model can be expressed as:

2 $${M_T}{\ddot {X}_T}+{C_T}{\dot {X}_T}+{K_T}{X_T}={P_T}$$

where, ${X_T}$ is displacement vector of the track model; ${M_T}$ is mass matrix of the track model; ${C_T}$ is damping matrix of the track model; ${K_T}$is stiffness matrix of the track model; ${P_T}$ is load vector of the track model.

### Multi-scale track defect model

A multi-scale track defect model is introduced to simulate rail corrugation, fastener looseness, and CA mortar layer disengagement.

#### Simulation model of rail corrugation

Rail corrugation is a regular wear pattern on the top surface of the rail. The most direct way to simulate rail corrugation in the vehicle-track coupling model is to use a rail profile with wavy abrasion when calculating the geometric relationship of wheel-rail spatial contact. Setting wavy wear data in rail profile data can generate a large and complex amount of data for the model, making it difficult to process. Rail corrugation can be regarded as a periodic track irregularity^27^, expressed as:

3 $${Z_{cor}}(t)=\frac{1}{2}a\left[ {1 - \cos \left( {\frac{{2\pi vt}}{\lambda }} \right)} \right]{\text{ }}\left( {0 \leqslant t \leqslant \frac{L}{v}} \right)$$

where, *a* is the depth of the rail corrugation; $\lambda$ is the wavelength of the rail corrugation; *L* is the length of the rail corrugation distributed on the rail; *v* is the running speed of the vehicle.

The simulation of rail corrugation is carried out using the superposition principle of track irregularity. Overlay the periodic rail corrugation with the random track irregularity, convert it into the rail position offset, and then use it as the excitation for the vehicle track coupling model. The total vertical offset of the rail is

4 $$\Delta {Z_{rail}}=\Delta {z_{random}}+{Z_{cor}}$$

where, $\Delta {z_{random}}$ is the vertical random displacement of the rail obtained from the track irregularity spectrum.

The rail corrugation involves two parameters, and different combinations of wavelength and depth create complex and varied working conditions. The irregularity superposition method can quickly simulate rail corrugation at various scales.

#### Refined model of fastener system

The preloaded force of the fastener system effectively indicates the looseness of the fastener, and its magnitude is directly proportional to the degree of fastener looseness. To accurately simulate fastener looseness and establish a refined fastener system model, the vertical force of the fastener system is

5 $${F_{fz}}(t)={F_{sr1}}(t)+{F_{sr2}}(t) - {F_{rp}}(t)$$

where, ${F_{sr1}}(t)$ and ${F_{sr2}}(t)$ are the clamping forces of the spring rods respectively, and ${F_{rp}}(t)$ is the supporting forces of the rail pad, which is calculated by the preloaded force and dynamic deformation^28^.

**Fig. 2:**
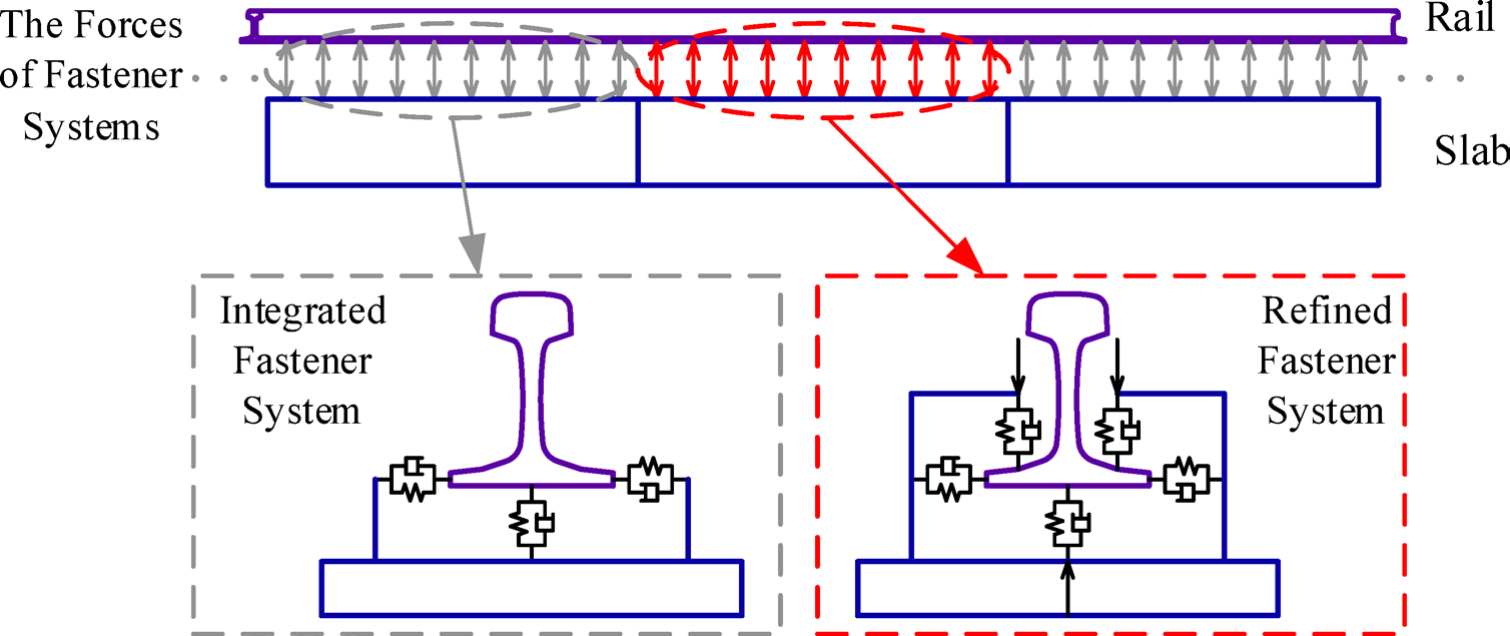
Multi-scale fastener model for fastener looseness simulation.

In each step of the numerical integration of the model, calculating the interaction force based on the relative position of the rail and slab in the refined fastener system model takes a long time, which slows down the model solving. To enhance simulation efficiency, a multi-scale fastener model with integrated and refined fastener systems is adopted, as shown in Fig. 2. The fasteners used to simulate looseness adopt refined fastener models, while fasteners at other positions use integrated fastener models.

When the vehicle speed is 300 km/h and the integration step is 0.0001s, the simulation time of vehicle-track coupling models with different fastener models is shown in Table 1. The simulation results indicate that the multi-scale fastener model can significantly improve simulation efficiency when simulating fastener looseness.

**Table 1:** The simulation time of the vehicle-track model with different fastener models.

Fastener System	20 Slabs	30 Slabs	40 Slabs	50 Slabs
Integrated Fastener Model	1219s	2518s	5389s	8543s
Refined Fastener Model	2321s	4878s	9556s	15085s
Multi-scale Fastener Model	1238s	2543s	5443s	8547s

#### Discrete model of CA mortar layer

**Fig. 3:**
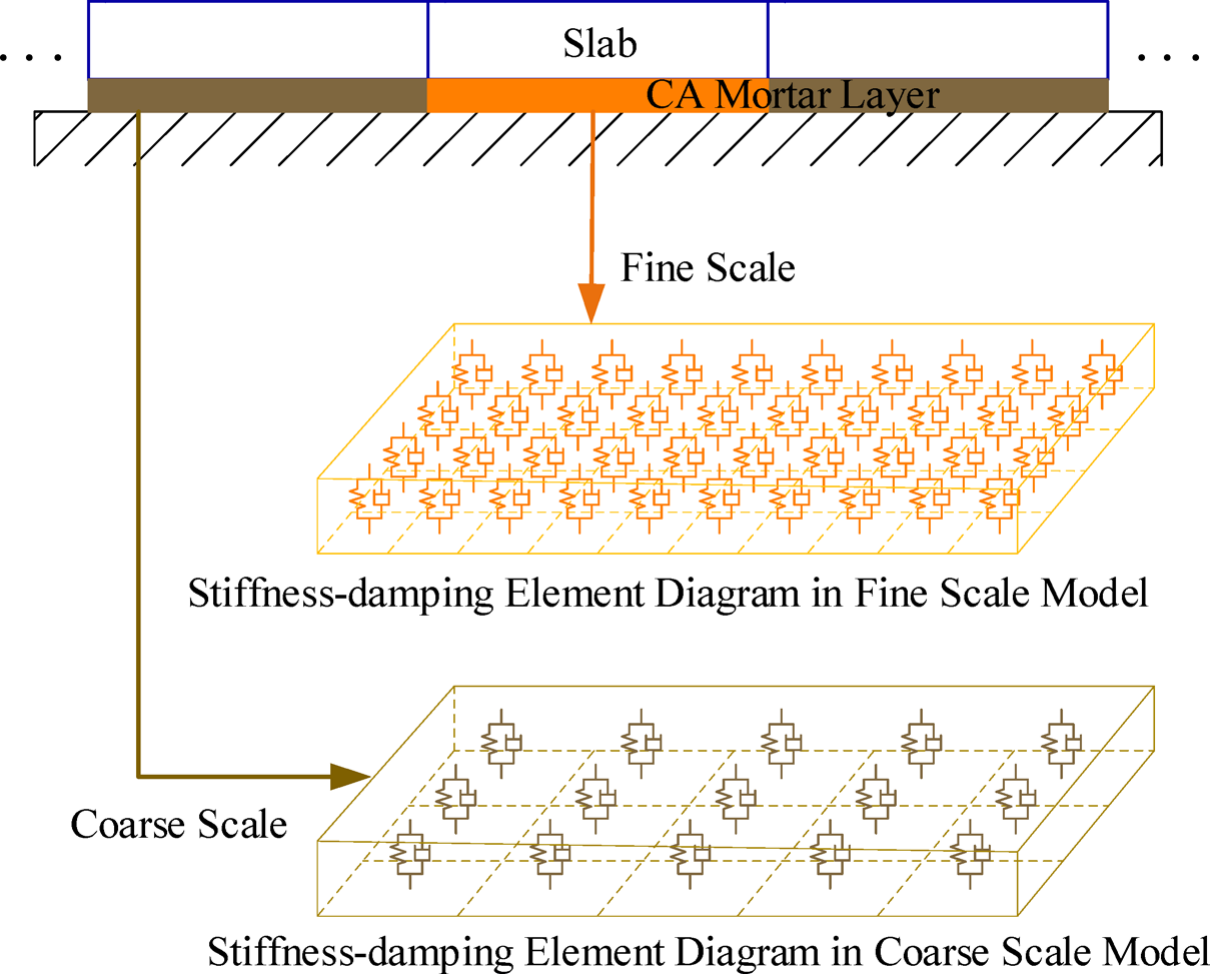
Multi-scale CA mortar layer model for CA mortar disengagement.

The slab ballastless track model employs the force of discrete support elements to represent the CA mortar layer. The CA mortar discretization model enables the simulation of voids within the CA mortar layer. The large number of discrete elements in the CA mortar layer can cause an increase model parameters and construction time, but the size and position of CA mortar disengagement simulation are more accurate. On the contrary, having fewer discrete elements in the CA mortar layer simplifies the model and speeds up the simulation calculation process, but it makes accurately simulating the CA mortar disengagement more difficult. The combination of coarse-scale and fine-scale models can improve the speed of establishing the slab ballastless track model, as shown in Fig. 3. Compared to the CA mortar layer in the normal position, the CA mortar layer used to simulate disengagement is divided into more discrete elements.

The construction time of the vehicle-track coupling model with different scale CA mortar layer models is shown in Table 2. The coarse-scale CA mortar layer discrete model in the simulation is divided into 20 units lengthwise and 10 units widthwise. The fine-scale CA mortar layer discrete model is divided into 100 units lengthwise and 40 units widthwise. The simulation results show that the multi-scale CA mortar layer model can reduce the modeling time of the vehicle-track coupling model.

**Table 2:** The construction time of the vehicle-track model with different CA mortar models.

CA Mortar Layer	20 Slabs	30 Slabs	40 Slabs	50 Slabs
Coarse Scale Model	0.177s	0.386s	0.691s	1.074s
Fine Scale Model	0.223s	0.448s	0.756s	1.207s
Multi-scale Model	0.179s	0.391s	0.698s	1.081s

### Segmental numerical solution method

According to the vehicle track coupling dynamics theory, the vehicle model and track model can be combined into a large-scale nonlinear differential equation system, meaning that Eq. (1) and Eq. (2) can be combined and represented as:

6 $$\left[ {\begin{array}{*{20}{c}} {{M_V}}&0 \\ 0&{{M_T}} \end{array}} \right]\left[ {\begin{array}{*{20}{c}} {{{\ddot {X}}_V}} \\ {{{\ddot {X}}_T}} \end{array}} \right]+\left[ {\begin{array}{*{20}{c}} {{C_V}}&0 \\ 0&{{C_T}} \end{array}} \right]\left[ {\begin{array}{*{20}{c}} {{{\dot {X}}_V}} \\ {{{\dot {X}}_T}} \end{array}} \right]+\left[ {\begin{array}{*{20}{c}} {{K_V}}&0 \\ 0&{{K_T}} \end{array}} \right]\left[ {\begin{array}{*{20}{c}} {{X_V}} \\ {{X_T}} \end{array}} \right]=\left[ {\begin{array}{*{20}{c}} {{P_V}} \\ {{P_T}} \end{array}} \right]$$

Using the fast explicit time integration method^25^ to solve the model, predict the displacement and velocity for the next integration step of the system as follows:

7 $$\left\{ \begin{gathered} {X_{n+1}}={X_n}+{V_n} \cdot \Delta t+(1/2+\alpha ) \cdot {A_n} \cdot \Delta {t^2} - \alpha \cdot {A_{n - 1}} \cdot \Delta {t^2} \hfill \\ {V_{n+1}}={V_n}+(1+\gamma ) \cdot {A_n} \cdot \Delta t - \gamma \cdot {A_{n - 1}} \cdot \Delta t \hfill \\ \end{gathered} \right.$$

where, *X*, *V* and *A *are the displacement, velocity, and acceleration of the system, respectively; $\Delta t$ is the integration step size;$\alpha$ and $\gamma$are integral control parameters, respectively.

When solving the integration of the high-speed vehicle and slab ballastless track coupling dynamic model, in each integration period, the acceleration increment, velocity increment, and displacement increment all meet the dynamic equilibrium condition of Eq. (6).

**Fig. 4:**
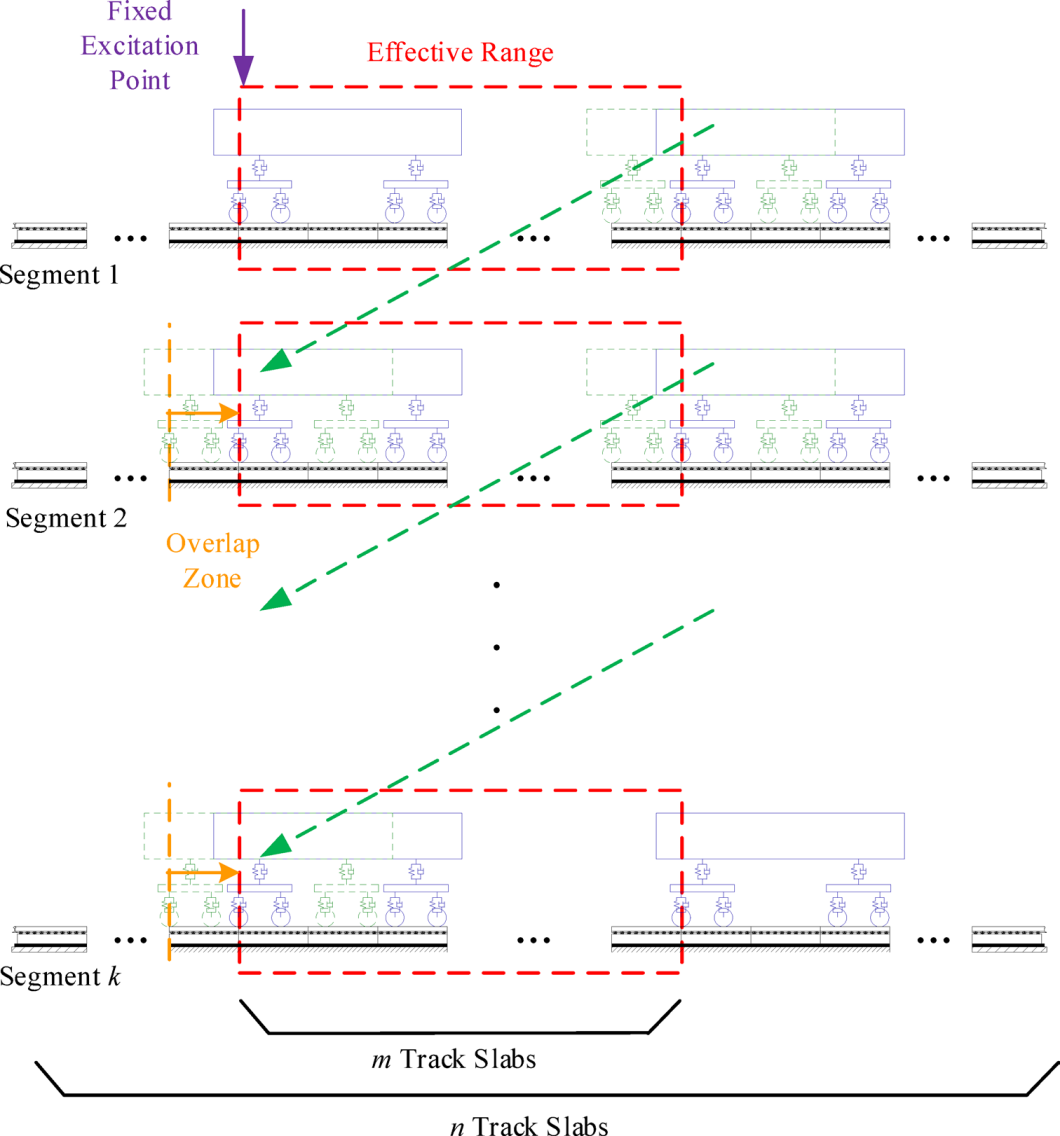
Segmented solution process of the vehicle-track coupling model.

The degree of freedom of the slab ballastless track model depends on the length of the track. Extending the track length significantly increases the degree of freedom in the vehicle-track coupling model. Solving the vehicle-track coupling model with a long track involves performing high-dimensional matrix calculations, which require a computer with high-performance CPUs and plenty of memory. Improving the efficiency of solving the vehicle-track coupling model on an ordinary computer will help gather a large amount of vehicle system vibration response data for researching track defect detection algorithms. Therefore, a numerical solution method named the segment-by-segment method was introduced to solve the vehicle-track coupling model. The segmented solution process of the vehicle-track coupling model is shown in Fig. 4.

The dynamic response of the vehicle at the end of the current segment track (Segment *k*) is considered the same as the dynamic response of the vehicle at the start of the next segment track (Segment *k* + 1), expressed as

8 $$\left\{ \begin{gathered} X_{V}^{{k+1}}({t_s})=X_{V}^{k}({t_e}) \hfill \\ \dot {X}_{V}^{{k+1}}({t_s})=\dot {X}_{V}^{k}({t_e}) \hfill \\ \ddot {X}_{V}^{{k+1}}({t_s})=\ddot {X}_{V}^{k}({t_e}) \hfill \\ \end{gathered} \right.$$

where, ${t_e}$ represents the time when the vehicle reaches the end position of the current segment track; ${t_s}$ represents the time when the vehicle reaches the starting position of the next segment track.

The rail model adopts the Ritz method to introduce mode functions and mode time coordinates of the rail. Due to the vehicle position changes between the two track segments, it is not possible to directly use the mode time coordinates of the rail at the end of the current segment (Segment *k*) as the initial mode time coordinates for the start of next segment (Segment *k* + 1). When the vehicle interacts with the track, the part of the track far away from the vehicle position has almost no impact on the system. The vibration response of the rail within a certain range of the end of the current track segment and the start of the next segment can be made equal, as expressed as

9 $$\left\{ \begin{gathered} Y_{r}^{{k+1}}({X_s},{t_s})=Y_{r}^{k}({X_e},{t_e}) \hfill \\ \dot {Y}_{r}^{{k+1}}({X_s},{t_s})=\dot {Y}_{r}^{k}({X_e},{t_e}) \hfill \\ \ddot {Y}_{r}^{{k+1}}({X_s},{t_s})=\ddot {Y}_{r}^{k}({X_e},{t_e}) \hfill \\ Z_{r}^{{k+1}}({X_s},{t_s})=Z_{r}^{k}({X_e},{t_e}) \hfill \\ \dot {Z}_{r}^{{k+1}}({X_s},{t_s})=\dot {Z}_{r}^{k}({X_e},{t_e}) \hfill \\ \ddot {Z}_{r}^{{k+1}}({X_s},{t_s})=\ddot {Z}_{r}^{k}({X_e},{t_e}) \hfill \\ \phi _{r}^{{k+1}}({X_s},{t_s})=\phi _{r}^{k}({X_e},{t_e}) \hfill \\ \dot {\phi }_{r}^{{k+1}}({X_s},{t_s})=\dot {\phi }_{r}^{k}({X_e},{t_e}) \hfill \\ \ddot {\phi }_{r}^{{k+1}}({X_s},{t_s})=\ddot {\phi }_{r}^{k}({X_e},{t_e}) \hfill \\ \end{gathered} \right.$$

where, ${Y_r}$, ${Z_r}$ and ${\phi _r}$ are rail lateral, vertical and torsional displacements, respectively; ${X_s}$ is the coordinate sequence of the rail position within a certain range of the start position in each segment track; ${X_e}$ is the coordinate sequence of the rail position within a certain range of the end position in each segment track.

According to the modal superposition principle of the Ritz method, Eq. (9) can be converted into

10 $$\left\{ \begin{gathered} Y{M_s} \cdot X_{{ry}}^{{k+1}}({t_s})=Y{M_e} \cdot X_{{ry}}^{k}({t_e}) \hfill \\ Y{M_s} \cdot \dot {X}_{{ry}}^{{k+1}}({t_s})=Y{M_e} \cdot \dot {X}_{{ry}}^{k}({t_e}) \hfill \\ Y{M_s} \cdot \ddot {X}_{{ry}}^{{k+1}}({t_s})=Y{M_e} \cdot \ddot {X}_{{ry}}^{k}({t_e}) \hfill \\ Z{M_s} \cdot X_{{rz}}^{{k+1}}({t_s})=Z{M_e} \cdot X_{{rz}}^{k}({t_e}) \hfill \\ Z{M_s} \cdot \dot {X}_{{rz}}^{{k+1}}({t_s})=Z{M_e} \cdot \dot {X}_{{rz}}^{k}({t_e}) \hfill \\ Z{M_s} \cdot \ddot {X}_{{rz}}^{{k+1}}({t_s})=Z{M_e} \cdot \ddot {X}_{{rz}}^{k}({t_e}) \hfill \\ H{M_s} \cdot X_{{rt}}^{{k+1}}({t_s})=H{M_e} \cdot X_{{rt}}^{k}({t_e}) \hfill \\ H{M_s} \cdot \dot {X}_{{rt}}^{{k+1}}({t_s})=H{M_e} \cdot \dot {X}_{{rt}}^{k}({t_e}) \hfill \\ H{M_s} \cdot \ddot {X}_{{rt}}^{{k+1}}({t_s})=H{M_e} \cdot \ddot {X}_{{rt}}^{k}({t_e}) \hfill \\ \end{gathered} \right.$$

where, ${X_{ry}}$, ${X_{rz}}$ and ${X_{rt}}$ are lateral, vertical and torsional mode time coordinates of rail, respectively; $Y{M_s}$, $Z{M_s}$ and $H{M_s}$ are vertical and torsional mode shape matrices of rail at the initial position in each segment track, respectively; $Y{M_e}$, $Z{M_e}$ and $H{M_e}$ are vertical and torsional mode shape matrices of rail at the end position in each segment track, respectively.

Similarly, the dynamic response of the slab at the end of the current segment track (Segment *k*) is regarded as the dynamic response of the slab at the start of next segment track (Segment *k* + 1), expressed as

11 $$\left\{ \begin{gathered} X_{{TS,{N_s}-{N_l}}}^{{k+1}}({t_s})=X_{{TS,{N_e}-{N_l}}}^{k}({t_e}) \hfill \\ \dot {X}_{{TS,{N_s}-{N_l}}}^{{k+1}}({t_s})=\dot {X}_{{TS,{N_e}-{N_l}}}^{k}({t_e}) \hfill \\ \ddot {X}_{{TS,{N_s}-{N_l}}}^{{k+1}}({t_s})=\ddot {X}_{{TS,{N_e}-{N_l}}}^{k}({t_e}) \hfill \\ \cdots \hfill \\ X_{{TS,{N_s}}}^{{k+1}}({t_s})=X_{{TS,{N_e}}}^{k}({t_e}) \hfill \\ \dot {X}_{{TS,{N_s}}}^{{k+1}}({t_s})=\dot {X}_{{TS,{N_e}}}^{k}({t_e}) \hfill \\ \ddot {X}_{{TS,{N_s}}}^{{k+1}}({t_s})=\ddot {X}_{{TS,{N_e}}}^{k}({t_e}) \hfill \\ \cdots \hfill \\ X_{{TS,{N_s}+{N_l}}}^{{k+1}}({t_s})=X_{{TS,{N_e}+{N_l}}}^{k}({t_e}) \hfill \\ \dot {X}_{{TS,{N_s}+{N_l}}}^{{k+1}}({t_s})=\dot {X}_{{TS,{N_e}+{N_l}}}^{k}({t_e}) \hfill \\ \ddot {X}_{{TS,{N_s}+{N_l}}}^{{k+1}}({t_s})=\ddot {X}_{{TS,{N_e}+{N_l}}}^{k}({t_e}) \hfill \\ \end{gathered} \right.$$

where, ${X_{TS,i}}$ represents the mode time coordinates of the *i*-th slab; ${N_s}$ is the slab number at the start position of the next segment track; ${N_e}$ is the slab number at the end position of the current segment track; ${N_l}$ is the number of slabs affected by vehicle excitation.

The segmented solution algorithm for the vehicle-track coupling model is presented in Table 3. Create an overlap zone before the effective range of each segment track to prevent discontinuities in the segmental solution results.

**Table 3:** Segmented solution algorithm for the vehicle-track coupling model.

Algorithm: Segmental Numerical Solution Method of Vehicle-track Coupling Model	
**Step 1: Parameter Setting**	
a.	**START**
b.	Set the length of each segment track and effective range
c.	Set the range of overlap zone
**Step 2: Model Building**	
d.	Construct mass matrix, damping matrix, and stiffness matrix according to Eq. (6)
**Step 3: Stable Initial Value Calculation**	
e.	Obtain stable initial values of the model through fixed-point excitation
**Step 4: Model Solution**	
f.	**DO**
g.	Using the fast explicit time integration method [25] to solve the current segment model
h.	Calculate the initial value of the vehicle dynamic response in the next segment of the model based on Eq. (8)
i.	Calculate the minimum norm solution of Eq. (10) to obtain the initial value of the rail dynamic response in the next segment of the model
j.	Calculate the initial value of the slab dynamic response in the next segment of the model according to Eq. (11)
k.	The track segment number $k=k+1$
l.	**END DO WHILE** The vehicle passes through all track segment
**Step 5: Data Storage**	
m.	Save displacement, velocity, and acceleration data of the vehicle and track
n.	**END**

The effectiveness of the proposed segmented solution method is confirmed by comparing the simulation time and the root mean square error (RMSE) of wheel-rail force across the three solution methods. The solution methods include the overall solution method (Method 1), the segmental solution method without an overlap zone (Method 2), and the segmental solution method (Method 3). Each segment track is made up of twenty slabs, with ten slabs in the middle of the track designated as the effective range and one slab designated as the overlap zone before the effective range. When the vehicle speed reaches 350 km/h, the calculation time of the vehicle-track coupling model and the RMSE of the wheel-rail force are shown in Table 4. Compared to directly solving the entire model, the segmental solution method significantly enhances the simulation efficiency of the vehicle-track coupling model. The accuracy of the solution results with overlap zone is higher, and the RMSE of the wheel-rail force simulation results is less than 0.3kN.

**Table 4:** Simulation time of the vehicle-track coupling model and the RMSE of wheel-rail force.

Track Length	Simulation Time of Method 1	Simulation Time of Method 2	RMSE of Wheel-rail Force (Use Method 2)	Simulation Time of Method 3	RMSE of Wheel-rail Force (Use Method 3)
130 m	3.45 h	1.17 h	0.20kN	1.83 h	0.15kN
195 m	7.61 h	1.98 h	0.26kN	2.15 h	0.19kN
260 m	12.91 h	2.25 h	0.27kN	2.46 h	0.21kN
325 m	22.01 h	2.55 h	0.29kN	2.78 h	0.23kN

## Dynamic response analysis of the vehicle system under track defects

Based on the multi-scale vehicle-track coupling model with track defects, this study further explores the sensitivity and variation laws of rail corrugation, fastener looseness, and CA mortar layer disengagement on the dynamic response of the vehicle system. It provides a theoretical foundation for decoupling track defect characteristics and developing a track defect detection algorithm based on wheelset vibration response. The following four cases are simulated, each involving different vehicle types and track irregularity spectra.

### Case 1

The parameters of CRH3 are used in the vehicle model, and the track random irregularity is simulated by the track irregularity Power Spectral Density (PSD) of Chinese high-speed railways.

### Case 2

The parameters of CRH3 are used in the vehicle model, and the track random irregularity is simulated by the low disturbance track irregularity PSD of Germany.

### Case 3

The parameters of CRH2 are used in the vehicle model, and the track random irregularity is simulated by the track irregularity PSD of Chinese high-speed railways.

### Case 4

The parameters of CRH2 are used in the vehicle model, and the track random irregularity is simulated by the low disturbance track irregularity PSD of Germany.

The dynamic parameters of the CRH2 vehicle are derived from Reference^29^ and others, while the dynamic parameters of the CRH3 vehicle are obtained from Reference^30^ and others. Currently, both types of vehicles are running on China’s high-speed railways. The carbody weight, suspension stiffness, and suspension damping of the two vehicle models vary, resulting in differences in vibration characteristics. The track irregularity PSD of Chinese high-speed railways and the low disturbance track irregularity PSD of Germany are two ways to express the random irregularity of high-speed railway^25^. The track irregularity value simulated using the track irregularity PSD of Chinese high-speed railways will be lower than that simulated using the low disturbance track irregularity PSD of Germany. Compared to the low disturbance track irregularity PSD of Germany, the track irregularity PSD of Chinese high-speed railways can be used to simulate the random irregularity of lines with better conditions. Therefore, the four cases, consisting of two vehicle types and two track irregularity spectra, can represent different working conditions, which helps enhance the realism and complexity of the data. Additionally, the dynamic parameters of the slab ballastless track are derived from Reference^30^, including the elastic modulus of the rail, the stiffness of the fastener system, the size of the track slab, and the elastic properties of CA mortar.

For the simulation of track defects, 28 kinds of rail corrugation (the depths are 0.04 mm, 0.05 mm, 0.06 mm, 0.07 mm, 0.08 mm, 0.09 mm and 0.1 mm, respectively; the wavelengths are 120 m, 130 mm, 140 mm and 150 mm, respectively), 15 kinds of fastener looseness (the number of fastener looseness ranges from 1 to 15), and 20 kinds of CA mortar disengagement (the void lengths range from 0.325 m to 6.5 m, with an interval of 0.325 m) are respectively set in the multi-scale track model. The vehicle speed ranges from 250 km/h to 350 km/h. Using the proposed segmental numerical solution method, each vehicle type can obtain 8400 sets of wheelset vibration acceleration data under rail corrugation, 4500 sets of wheelset vibration acceleration data under fastener looseness, and 6000 sets of wheelset vibration acceleration data under CA mortar layer disengagement.

### Effect of rail corrugation on the dynamic response of the vehicle wheelset

**Fig. 5:**
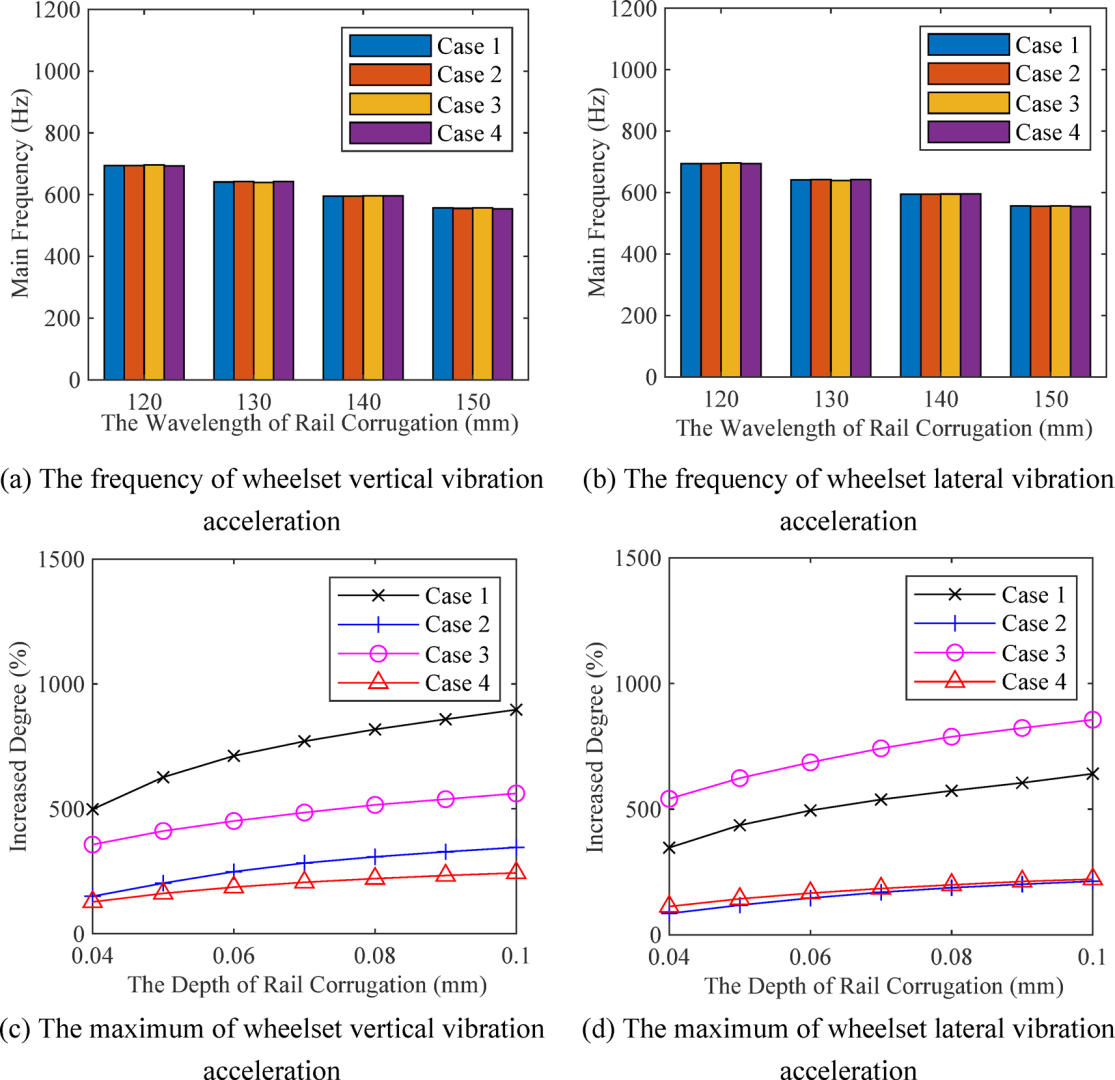
Effect of rail corrugation on wheelset dynamic response.

The operating speed of high-speed vehicles is 300 km/h, and the average values of the main frequencies of vertical vibration acceleration and lateral vibration acceleration of wheelsets are shown in Fig. 5(a) and (b). The main frequency of wheelset vibration acceleration matches the excitation frequency of rail corrugation. When the depth increases from 0.04 mm to 0.1 mm, the changes in vertical vibration acceleration and lateral vibration acceleration of the wheelset are shown in Fig. 5(c) and (d). Under different vehicle types and track random irregularity spectra, the vibration response of the wheelset increases as the rail corrugation depth grows. Due to variations in vehicle parameters and track irregularities, the maximum vibration acceleration and the degree of increase in wheelset vibration vary under different cases. Therefore, the simple threshold judgment methods cannot be used to detect the level of rail corrugation.

### Effect of fastener looseness on the dynamic response of the vehicle wheelset

As the number of fastener looseness increases from 1 to 15, the change in amplitude energy of the PSDs of the wheelset vibration acceleration is shown in Table 5. Under different vehicle types and track random irregularity spectra, as the number of fastener looseness increases, the amplitude energy change in the PSDs of wheelset vibration acceleration significantly increases. The magnitude difference in the energy variation of the PSD of wheelset vibration acceleration is significant, and multiple maximum values appear, indicating a nonlinear relationship between wheelset vibration acceleration and the number of fastener looseness. The change in PSD of vertical vibration acceleration of wheelsets is generally smaller than that of lateral acceleration, reflecting that fastener looseness has a greater impact on the lateral vibration of wheelsets than on vertical vibration. Therefore, when performing fastener looseness detection, it is crucial to focus on analysing fluctuations in the lateral vibration of the wheelset.

**Table 5:** Effect of the number of fastener looseness on wheelset dynamic response (Unit: %).

Number	Case 1 Vertical	Case 1 Lateral	Case 2 Vertical	Case 2 Lateral	Case 3 Vertical	Case 3 Lateral	Case 4 Vertical	Case4 Lateral
1	1.67	11.20	0.59	7.96	1.39	6.50	0.54	3.13
2	1.93	18.14	1.01	16.90	1.71	10.07	0.96	5.70
3	2.44	20.88	1.36	35.38	2.04	14.17	1.40	9.06
4	2.95	21.33	1.95	65.92	2.44	20.90	2.18	14.25
5	4.17	23.10	2.15	92.24	3.42	25.64	2.26	23.70
6	7.47	30.32	2.60	121.44	5.96	32.72	2.69	46.91
7	8.17	29.10	4.14	148.11	6.73	43.26	3.69	76.49
8	7.41	67.05	4.93	106.16	6.00	42.48	4.71	236.43
9	15.54	3.42 × 10^3^	4.72	230.88	11.03	1.42 × 10^3^	5.04	133.73
10	9.49	27.55	6.06	138.62	7.59	56.65	6.36	368.31
11	8.06	90.72	5.61	421.70	6.74	124.87	5.70	722.94
12	15.56	3.67 × 10^3^	7.32	274.04	10.93	362.51	6.32	442.46
13	41.25	5.39 × 10^6^	15.54	4.80 × 10^4^	18.37	1.95 × 10^6^	10.79	1.50 × 10^4^
14	83.33	4.33 × 10^7^	19.39	3.85 × 10^5^	29.53	7.71 × 10^6^	14.50	6.44 × 10^5^
15	121.04	9.47 × 10^6^	21.81	1.79 × 10^5^	40.39	1.52 × 10^6^	14.90	1.33 × 10^6^

### Effect of CA mortar layer disengagement on the dynamic response of the vehicle wheelset

**Fig. 6:**
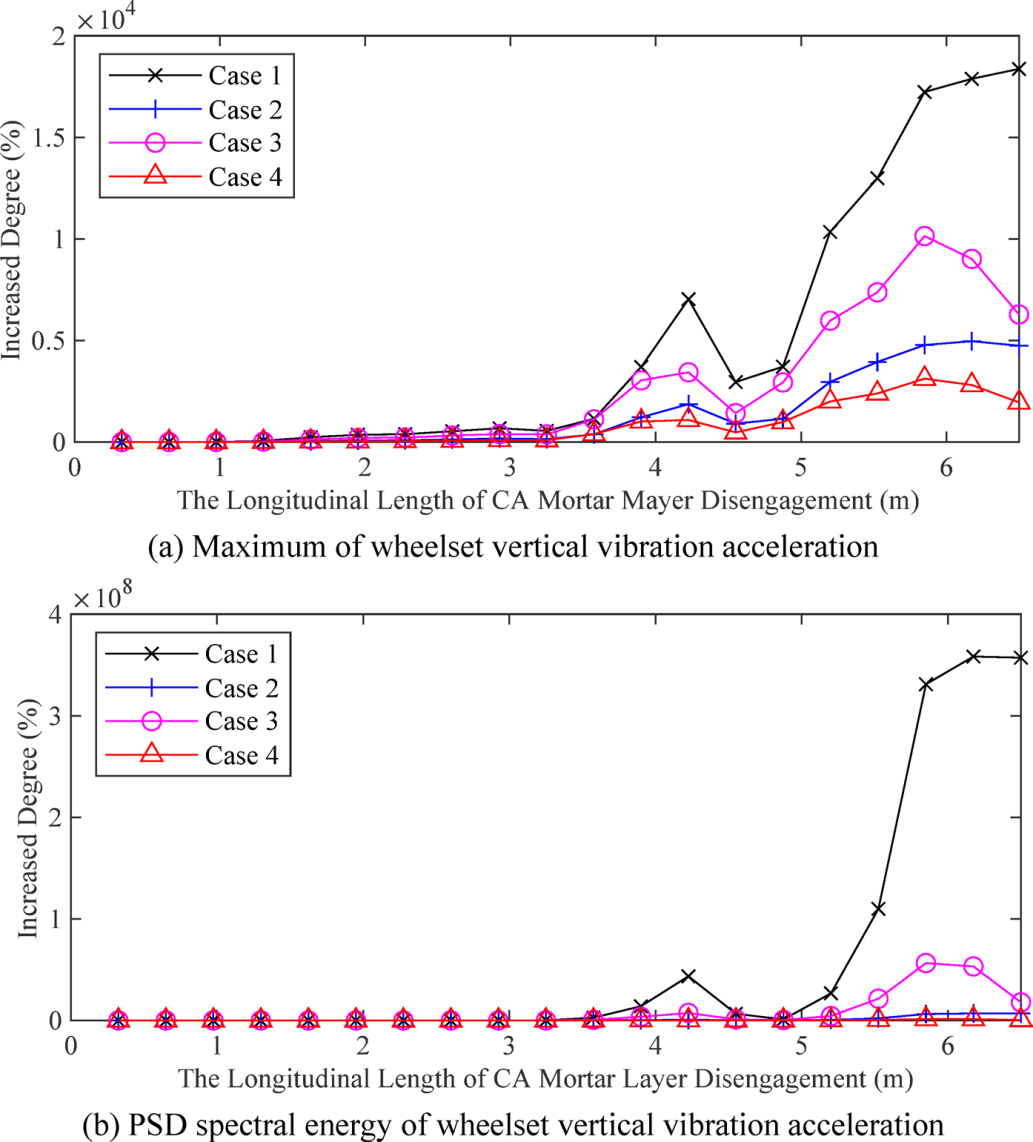
Effect of CA mortar layer disengagement length on wheelset vertical dynamic response.

According to the CA mortar layer model, it is known that the voids in the CA mortar layer will influence the vertical vibration response of the vehicle and track system. When the longitudinal disengagement length of the CA mortar increases from 0.325 m to 6.5 m, the changes in the amplitude of the vertical vibration acceleration of the wheelset and the amplitude energy variations of its PSD are shown in Fig. 6. Under different vehicle types and track random irregularity spectra, the vibration response of the wheelset increases as the disengagement length of the CA mortar layer grows. The impact of CA mortar disengagement with a void length of less than 0.65 m on the vibration response of wheelsets is relatively small. Owing to differences in vehicle parameters and track irregularity, the maximum value and degree of increase in wheelset vibration acceleration vary under different cases. Therefore, when conducting CA mortar layer disengagement detection, it is essential to focus on analysing the deviations in the vertical vibration of the wheelset.

## Track defects characteristic decoupling algorithm based on prior knowledge

The excitation forms and influence levels of rail corrugation, fastener looseness, CA mortar disengagement, and track irregularity are various, making it difficult for a single signal processing method to extract characteristic information that reflects track defects comprehensively. Based on the analysis of the vibration response of the vehicle system under the influence of track defects, a track defect characteristic decoupling algorithm is proposed, utilizing prior knowledge and multiple signal processing techniques. Using prior knowledge of how track defects affect wheelset vibration responses, signal decomposition and reconstruction are performed with methods such as Ensemble Empirical Mode Decomposition (EEMD), Wavelet Packet Decomposition (WPD), and time-frequency analysis. These methods help obtain sensitive feature data for various track defects, reduce the influence of track irregularities, and enable feature extraction of wheelset vibration signals caused by defects like rail corrugation, fastener looseness, and CA mortar disengagement.

### Decoupling of rail corrugation characteristics

**Fig. 7:**
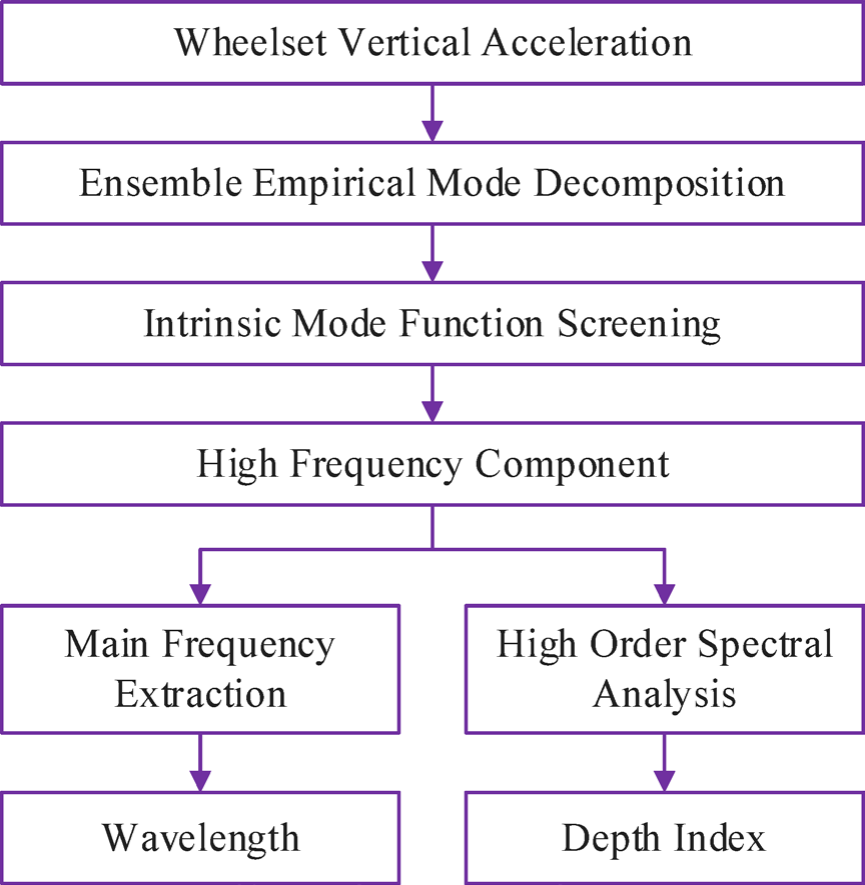
Schematic diagram of rail corrugation characteristics decoupling.

According to the vibration response law of wheelsets under rail corrugation, it is known that high-frequency components caused by rail corrugation appear in the vertical vibration response signal of wheelsets. Thus, the core of the decoupling algorithm for rail corrugation characteristics is to extract high-frequency signals from the vertical vibration acceleration signal of wheelsets. To address the decoupling problem of rail corrugation characteristics in the composite vibration response of wheelsets, a corrugation wavelength estimation and corrugation depth feature extraction algorithm based on EEMD and high-order spectral analysis is proposed. The schematic diagram of the decoupling algorithm for rail corrugation characteristics is shown in Fig. 7.

The wavelength estimation for the rail corrugation wavelength is based on EEMD. First, the vertical vibration response signal of the wheelset is decomposed with EEMD, followed by intrinsic mode function analysis and screening. Automatically select the parameters of EEMD based on the extreme point distribution index and orthogonality index^27^, including choosing the appropriate number of ensemble members and the optimal white noise amplitude. Next, high-frequency components related to corrugation excitation are extracted to identify the frequency corresponding to the corrugation wavelength. Finally, the corrugation wavelength is calculated based on the vehicle operating speed, expressed as

12 $$\lambda =\frac{{1000V}}{{3.6{f_m}}}$$

where, *V* is the vehicle speed (km/h); ${f_m}$ is the main frequency of the high frequency component of the wheelset vibration response (Hz).

Compared to simple spectral analysis, high-order spectral analysis can further minimize the impact of track random irregularities and other excitation frequencies, while emphasizing the amplitude and energy characteristics of high-frequency components caused by rail corrugation excitation in the wheelset vibration acceleration signal. Using high-order spectral analysis effectively helps decouple and extract the depth features of rail corrugation. The most commonly used high-order spectral estimation method is the direct method. Firstly, estimate the high-order cumulative function, and then calculate the high-order spectrum through the discrete Fourier transform. The frequency domain of the third-order cumulant of wheelset vibration response reconstructed based on high-frequency components is

13 $$B({f_1},{f_2})=E\left[ {X({f_1})X({f_2}){X^ * }({f_1}+{f_2})} \right]$$

where, $X( \cdot )$ represents the Fourier transform of the signal; * represents a conjugate complex number; $E( \cdot )$ represents expectation.

After estimating the wavelength of the corrugation, the high-order spectral estimation direct method is used to perform bispectrum analysis on the wheelset vibration response reconstructed based on high-frequency components, resulting in a discrete bispectrum matrix ***B***. The size of the bispectrum matrix ***B*** is *Q*×*Q*. In order to facilitate the characterization of the depth characteristics of rail corrugation, feature extraction was performed based on the bispectrum matrix ***B***. The expressions of the depth characteristics of the rail corrugation depth in the wheelset vibration response are shown in Table 6.

**Table 6:** Feature values of rail corrugation based on higher-order spectrum.

Feature Description	Expression
Average Value	${H_1}=\frac{1}{{{Q^2}}}\sum\limits_{{i=1}}^{Q} {\sum\limits_{{j=1}}^{Q} {\left| {B(i,j)} \right|} }$
Logarithmic Sum	${H_2}=\sum\limits_{{i=1}}^{Q} {\sum\limits_{{j=1}}^{Q} {\log \left( {\left| {B(i,j)} \right|} \right)} }$
Logarithmic Sum of Diagonal Values	${H_3}=\sum\limits_{{i=1}}^{Q} {\log \left( {\left| {B(i,i)} \right|} \right)}$
First Moment of Diagonal Values	${H_4}=\sum\limits_{{i=1}}^{Q} {i \cdot \log \left( {\left| {B(i,i)} \right|} \right)}$
Second Moment of Diagonal Values	${H_5}=\sum\limits_{{i=1}}^{Q} {{{(i - {H_4})}^2} \cdot \log \left( {\left| {B(i,i)} \right|} \right)}$
Central Moment	${H_6}=\sum\limits_{{i=1}}^{Q} {\sum\limits_{{j=1}}^{Q} {\sqrt {{i^2} - {j^2}} \cdot \left| {B(i,i)} \right|} }$
Entropy	$$\begin{gathered} {H_7}= - \sum\limits_{{i=1}}^{Q} {\sum\limits_{{j=1}}^{Q} {{P_1}(i,j) \cdot \log \left( {{P_1}(i,j)} \right)} } \hfill \\ \begin{array}{*{20}{c}} {}&{}&{}&{} \end{array}{P_1}(i,j)={{\left| {B(i,j)} \right|} \mathord{\left/ {\vphantom {{\left| {B(i,j)} \right|} {\sum\limits_{{i=1}}^{Q} {\sum\limits_{{j=1}}^{Q} {\left| {B(i,j)} \right|} } }}} \right. \kern-0pt} {\sum\limits_{{i=1}}^{Q} {\sum\limits_{{j=1}}^{Q} {\left| {B(i,j)} \right|} } }} \hfill \\ \end{gathered}$$
Square Entropy	$$\begin{gathered} {H_8}= - \sum\limits_{{i=1}}^{Q} {\sum\limits_{{j=1}}^{Q} {{P_2}(i,j) \cdot \log \left( {{P_2}(i,j)} \right)} } \hfill \\ \begin{array}{*{20}{c}} {}&{}&{}&{} \end{array}{P_2}(i,j)={{{{\left| {B(i,j)} \right|}^2}} \mathord{\left/ {\vphantom {{{{\left| {B(i,j)} \right|}^2}} {\sum\limits_{{i=1}}^{Q} {\sum\limits_{{j=1}}^{Q} {{{\left| {B(i,j)} \right|}^2}} } }}} \right. \kern-0pt} {\sum\limits_{{i=1}}^{Q} {\sum\limits_{{j=1}}^{Q} {{{\left| {B(i,j)} \right|}^2}} } }} \hfill \\ \end{gathered}$$
Cubic Entropy	$$\begin{gathered} {H_9}= - \sum\limits_{{i=1}}^{Q} {\sum\limits_{{j=1}}^{Q} {{P_3}(i,j) \cdot \log \left( {{P_3}(i,j)} \right)} } \hfill \\ \begin{array}{*{20}{c}} {}&{}&{}&{} \end{array}{P_3}(i,j)={{{{\left| {B(i,j)} \right|}^3}} \mathord{\left/ {\vphantom {{{{\left| {B(i,j)} \right|}^3}} {\sum\limits_{{i=1}}^{Q} {\sum\limits_{{j=1}}^{Q} {{{\left| {B(i,j)} \right|}^3}} } }}} \right. \kern-0pt} {\sum\limits_{{i=1}}^{Q} {\sum\limits_{{j=1}}^{Q} {{{\left| {B(i,j)} \right|}^3}} } }} \hfill \\ \end{gathered}$$

To further quantify the depth characteristics of rail corrugation, the depth characteristic index (*DCI*) is defined based on nine extracted feature values to distinguish the depth characteristics of rail corrugation. The *DCI* is defined as

14 $$DCI=\sqrt {\sum\limits_{{k=1}}^{9} {H_{k}^{2}} }$$

**Fig. 8:**
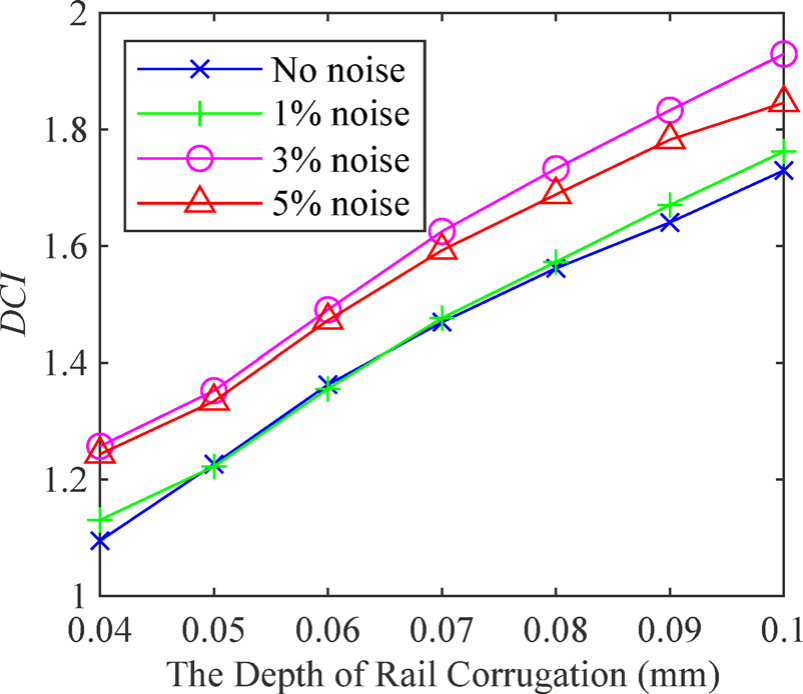
The depth index of rail corrugation.

White noise at 1%, 3%, and 5% amplitudes was added to the data to enhance its diversity and authenticity. The average values of the four corrugation wavelength estimates are 120.40 mm, 130.03 mm, 139.81 mm, and 149.47 mm. The results show that the wavelength estimation algorithm for rail corrugation, based on EEMD, can accurately determine the wavelength and remains unaffected by noise amplitude. The overall average estimation error is under 0.25%. The depth characteristic indexes of rail corrugation are shown in Fig. 8. The *DCI* monotonically rises as the depth of rail corrugation increases. The variation trend of the corrugation depth characteristic index *DCI* is consistent under different noises. Therefore, the *DCI* created by the rail corrugation depth feature extraction algorithm based on high-order spectrum can quantitatively describe the characteristics of rail corrugation depth and achieve decoupling of rail corrugation features without being affected by noise.

### Decoupling of fastener looseness and CA mortar layer disengagement characteristics

**Fig. 9:**
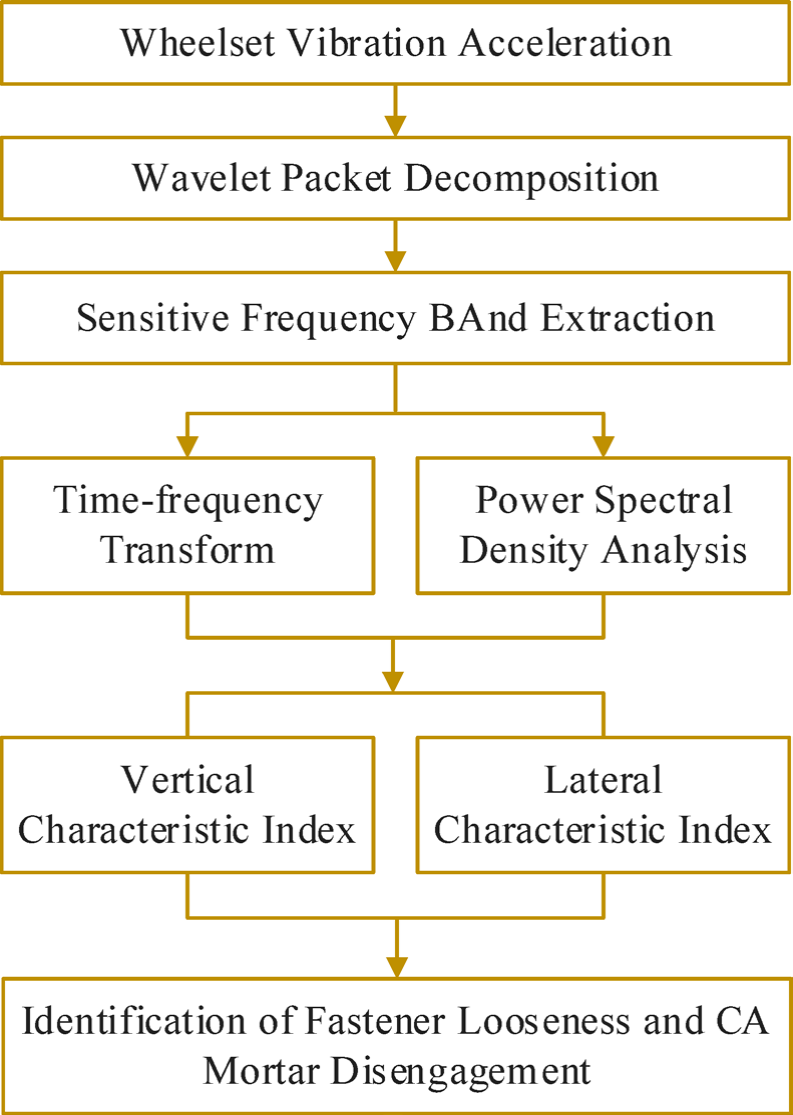
Schematic diagram of fastener looseness and CA mortar disengagement characteristics decoupling.

Compared to rail corrugation defects, the impact of fastener looseness and CA mortar layer disengagement on wheelset vibration response is relatively minor, and there is no specific excitation frequency. The effect of fastener looseness on the vertical vibration response of the wheelset is less than its impact on the lateral vibration response. Conversely, the effect of CA mortar layer disengagement on the vertical vibration response of the wheelset is greater than its effect on the lateral vibration response. Therefore, the main idea of the decoupling algorithm for the characteristics of fastener looseness and CA mortar layer disengagement is to identify the difference between how these two defects affect the vertical and lateral vibration responses of the wheelset. A feature extraction algorithm based on PSD analysis, WPD, and time-frequency transform is proposed to address the decoupling problem of fastener looseness and CA mortar disengagement characteristics in the wheelset vibration response, as shown in Fig. 9. This algorithm conducts sensitive frequency band analysis and feature extraction on the vertical and lateral vibration acceleration signals of the wheelset, and creates defect indexes to differentiate and characterize the defect features of fastener looseness and CA mortar layer disengagement.

Firstly, it is necessary to analyse the sensitive frequency range of the wheelset vibration response signal for fastener looseness and CA mortar layer disengagement to distinguish their characteristics accurately. According to the PSD analysis of wheelset vibration acceleration, it is observed that when the vehicle runs at high speed with minor defects, the effects of fastener looseness and CA mortar layer disengagement on the lateral and vertical vibration responses of the wheelset are mainly within the 0–1000 Hz range. When the defect severity increases, these issues influence the entire frequency range of the wheelset lateral vibration and wheelset vertical vibration. Considering different speeds and operating conditions, the sensitive frequency band for the wheelset lateral and vertical vibrations related to fastener looseness and CA mortar layer disengagement is determined to be 5–2000 Hz.

Define a PSD variation index of the vertical vibration acceleration signal of the wheelset (*EI*_*V*_), which is used to describe the sensitivity of the wheelset vertical vibration response to track defects and also reflects the influence of defects on the wheelset vertical vibration response. The expression of *EI*_*V*_ is

15 $$E{I_V}=\frac{{\left| {\sum\limits_{{k=1}}^{N} {P_{k}^{V}} - \sum\limits_{{k=1}}^{N} {\tilde {P}_{k}^{V}} } \right|}}{{\sum\limits_{{k=1}}^{N} {\tilde {P}_{k}^{V}} }} \times 100\%$$

where, ${P^V}$ represents the PSD of the wheelset vertical vibration response; ${\tilde {P}^V}$ represents the PSD of the wheelset vertical vibration response under normal condition.

Similarly, define a PSD variation index of the lateral vibration acceleration signal of the wheelset (*EI*_*L*_), which is used to describe the sensitivity of the wheelset lateral vibration response to track defects and also reflects the influence of defects on the wheelset lateral vibration response. The expression of *EI*_*L*_ is

16 $$E{I_L}=\frac{{\left| {\sum\limits_{{k=1}}^{N} {P_{k}^{L}} - \sum\limits_{{k=1}}^{N} {\tilde {P}_{k}^{L}} } \right|}}{{\sum\limits_{{k=1}}^{N} {\tilde {P}_{k}^{L}} }} \times 100\%$$

where, ${P^L}$ represents the PSD of the wheelset lateral vibration response; ${\tilde {P}^L}$ represents the PSD of the wheelset lateral vibration response under normal condition.

Secondly, the signature signals within sensitive frequency bands are extracted from the wheelset vibration acceleration signal using WPD. The Meyer function is selected as the wavelet basis function for the wavelet packet transform because its waveform resembles the vibration response of the wheelset caused by fastener looseness and CA mortar layer disengagement. The time interval of the wheelset vibration acceleration data corresponds to the step size of the numerical integration of the vehicle track coupling model, with a sampling frequency of 10 kHz. The WPD is set to a level of 10, dividing the wheelset vibration response signal into 1024 frequency bands, each with a bandwidth of 4.88 Hz, satisfying the requirement to extract sensitive frequency bands from 5 to 2000 Hz. The signal components are organized by frequency, and only those within the sensitive frequency bands are chosen for extraction. These components are then used to reconstruct the acceleration signal of the wheelset vibration response.

Thirdly, after obtaining the acceleration signal of the wheelset vibration response that only contains sensitive frequency bands, the WVD time-frequency transform is used to extract features of the wheelset vibration response. The time-frequency representation of the wheelset vibration response is

17 $$W(t,f)=\int_{{ - \infty }}^{{+\infty }} {{x^*}\left( {t - \frac{1}{2}\tau } \right)x\left( {t - \frac{1}{2}\tau } \right){e^{ - i2\pi f\tau }}d\tau }$$

A time-frequency matrix ***W*** with a size of *m*×*n* is obtained by performing time-frequency transformation on the wheelset vibration acceleration signal after wavelet packet decomposition and reconstruction, which can reflect the relationship between signal frequency and time. The expressions of the feature values of the track defects base time-frequency matrix are shown in Table 7.

**Table 7:** Feature values of track defects based on time-frequency matrix.

Feature Description	Expression
Small Time Dominance	${T_1}={{\sum\limits_{{i=1}}^{m} {\sum\limits_{{j=1}}^{n} {\frac{{{W_{ij}}}}{{{j^2}}}} } } \mathord{\left/ {\vphantom {{\sum\limits_{{i=1}}^{m} {\sum\limits_{{j=1}}^{n} {\frac{{{W_{ij}}}}{{{j^2}}}} } } {\sum\limits_{{i=1}}^{m} {\sum\limits_{{j=1}}^{n} {{W_{ij}}} } }}} \right. \kern-0pt} {\sum\limits_{{i=1}}^{m} {\sum\limits_{{j=1}}^{n} {{W_{ij}}} } }}$
Big Time Dominance	${T_2}={{\sum\limits_{{i=1}}^{m} {\sum\limits_{{j=1}}^{n} {{j^2} \cdot {W_{ij}}} } } \mathord{\left/ {\vphantom {{\sum\limits_{{i=1}}^{m} {\sum\limits_{{j=1}}^{n} {{j^2} \cdot {W_{ij}}} } } {\sum\limits_{{i=1}}^{m} {\sum\limits_{{j=1}}^{n} {{W_{ij}}} } }}} \right. \kern-0pt} {\sum\limits_{{i=1}}^{m} {\sum\limits_{{j=1}}^{n} {{W_{ij}}} } }}$
Frequency Distribution Inhomogeneity	${T_3}={{\sum\limits_{{i=1}}^{m} {{{\left[ {\sum\limits_{{j=1}}^{n} {{W_{ij}}} } \right]}^2}} } \mathord{\left/ {\vphantom {{\sum\limits_{{i=1}}^{m} {{{\left[ {\sum\limits_{{j=1}}^{n} {{W_{ij}}} } \right]}^2}} } {\sum\limits_{{i=1}}^{m} {\sum\limits_{{j=1}}^{n} {{W_{ij}}} } }}} \right. \kern-0pt} {\sum\limits_{{i=1}}^{m} {\sum\limits_{{j=1}}^{n} {{W_{ij}}} } }}$
Time Distribution Inhomogeneity	${T_4}={{\sum\limits_{{j=1}}^{n} {{{\left[ {\sum\limits_{{i=1}}^{m} {{W_{ij}}} } \right]}^2}} } \mathord{\left/ {\vphantom {{\sum\limits_{{j=1}}^{n} {{{\left[ {\sum\limits_{{i=1}}^{m} {{W_{ij}}} } \right]}^2}} } {\sum\limits_{{i=1}}^{m} {\sum\limits_{{j=1}}^{n} {{W_{ij}}} } }}} \right. \kern-0pt} {\sum\limits_{{i=1}}^{m} {\sum\limits_{{j=1}}^{n} {{W_{ij}}} } }}$
Energy	${T_5}=\sum\limits_{{i=1}}^{m} {\sum\limits_{{j=1}}^{n} {W_{{ij}}^{2}} }$
Frequency Mean	${T_6}=\sum\limits_{{i=1}}^{m} {i \cdot \left[ {\sum\limits_{{j=1}}^{n} {W_{{ij}}^{2}} } \right]}$
Time Mean	${T_7}=\sum\limits_{{j=1}}^{n} {j \cdot \left[ {\sum\limits_{{i=1}}^{m} {W_{{ij}}^{2}} } \right]}$
Frequency Variance	${T_8}=\sum\limits_{{i=1}}^{m} {\left[ {{{(i - {T_6})}^2} \cdot \sum\limits_{{j=1}}^{n} {{W_{ij}}} } \right]}$
Time Variance	${T_9}=\sum\limits_{{j=1}}^{n} {\left[ {{{(j - {T_7})}^2} \cdot \sum\limits_{{i=1}}^{m} {{W_{ij}}} } \right]}$
Correlation	${T_{10}}=\frac{1}{{{T_8} \cdot {T_9}}}\sum\limits_{{i=1}}^{m} {\sum\limits_{{j=1}}^{n} {(i - {T_6})(j - {T_7}){W_{ij}}} }$
Moment of Inertia	${T_{11}}=\sum\limits_{{i=1}}^{m} {\sum\limits_{{j=1}}^{n} {{{(i - j)}^2} \cdot {W_{ij}}} }$
Inverse Difference Moment	${T_{12}}=\sum\limits_{{i=1}}^{m} {\sum\limits_{{j=1}}^{n} {\frac{{{W_{ij}}}}{{1+{{(i - j)}^2}}}} }$

To quantitatively characterize the vibration response of wheels caused by fastener looseness and CA mortar disengagement, a vertical defect index and a lateral defect index are defined based on the extracted features from the time-frequency matrix and PSD variation index, respectively.

The vertical defect index (*VDI*) is defined as

18 $$VDI=\frac{1}{{13}}\sqrt {{{\left( {E{I_V}} \right)}^2}+\sum\limits_{{k=1}}^{{12}} {{{\left[ {\frac{{T_{k}^{V} - \tilde {T}_{k}^{V}}}{{\tilde {T}_{k}^{V}}}} \right]}^2}} }$$

where, ${T^V}$ represents the feature values of the wheelset vertical vibration response; ${\tilde {T}^V}$ represents the feature values of the wheelset vertical vibration response under normal condition.

The lateral defect index (*LDI*) is defined as

19 $$LDI=\frac{1}{{13}}\sqrt {{{\left( {E{I_L}} \right)}^2}+\sum\limits_{{k=1}}^{{12}} {{{\left[ {\frac{{T_{k}^{L} - \tilde {T}_{k}^{L}}}{{\tilde {T}_{k}^{L}}}} \right]}^2}} }$$

where, ${T^L}$ represents the feature values of the wheelset lateral vibration response; ${\tilde {T}^L}$ represents the feature values of the wheelset lateral vibration response under normal condition.

**Fig. 10:**
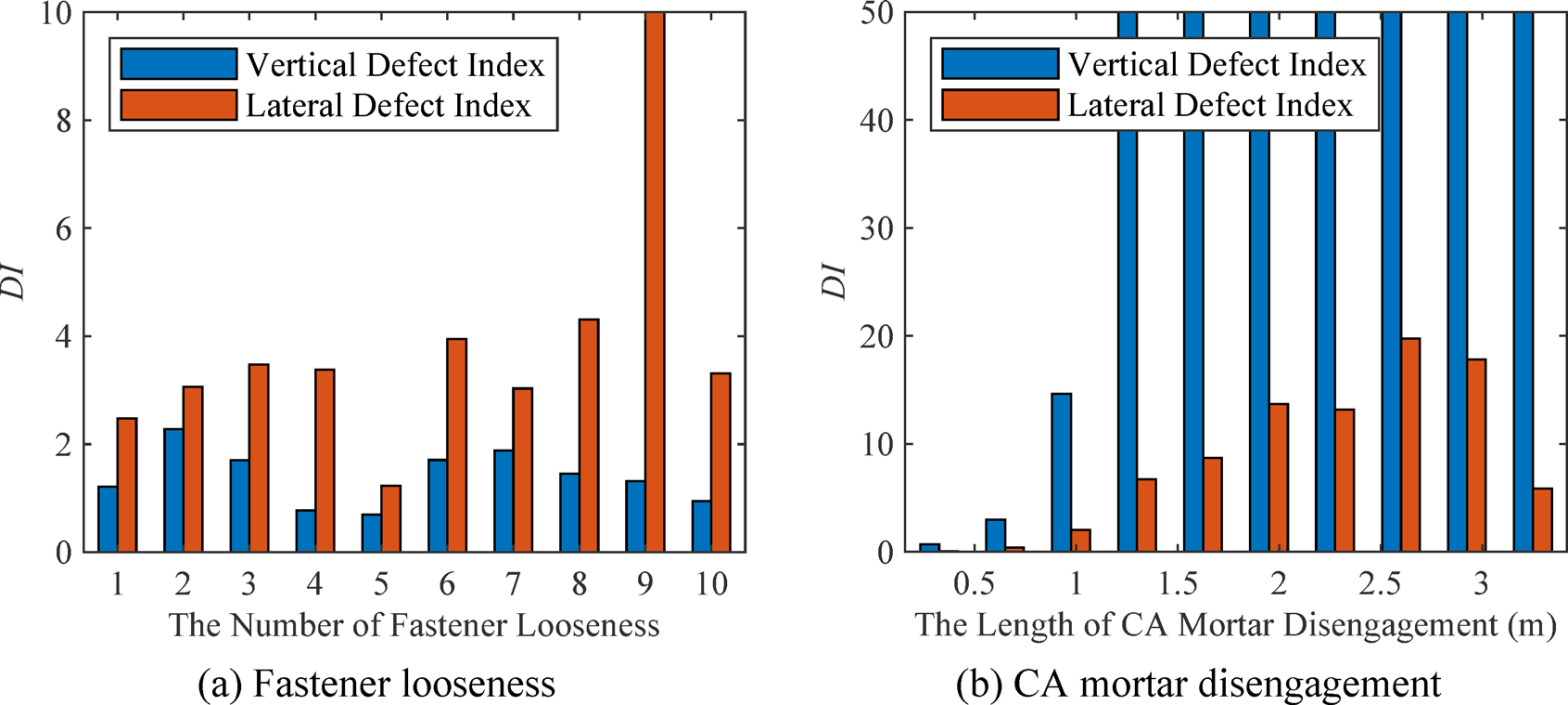
Defect index of wheelset vibration response under fastener looseness and CA mortar disengagement.

The defect indexes of wheelset vibration response under fastener looseness and CA mortar disengagement are shown in Fig. 10. The constructed *VDI* and *LDI* can describe and distinguish between fastener looseness and CA mortar layer disengagement. When there is fastener looseness on the track, the *VDI* is lower than the *LDI*. Conversely, when CA mortar disconnection occurs, the *VDI* is higher than the *LDI*.

## Multi-stage intelligent detection algorithm of track defects

**Fig. 11:**
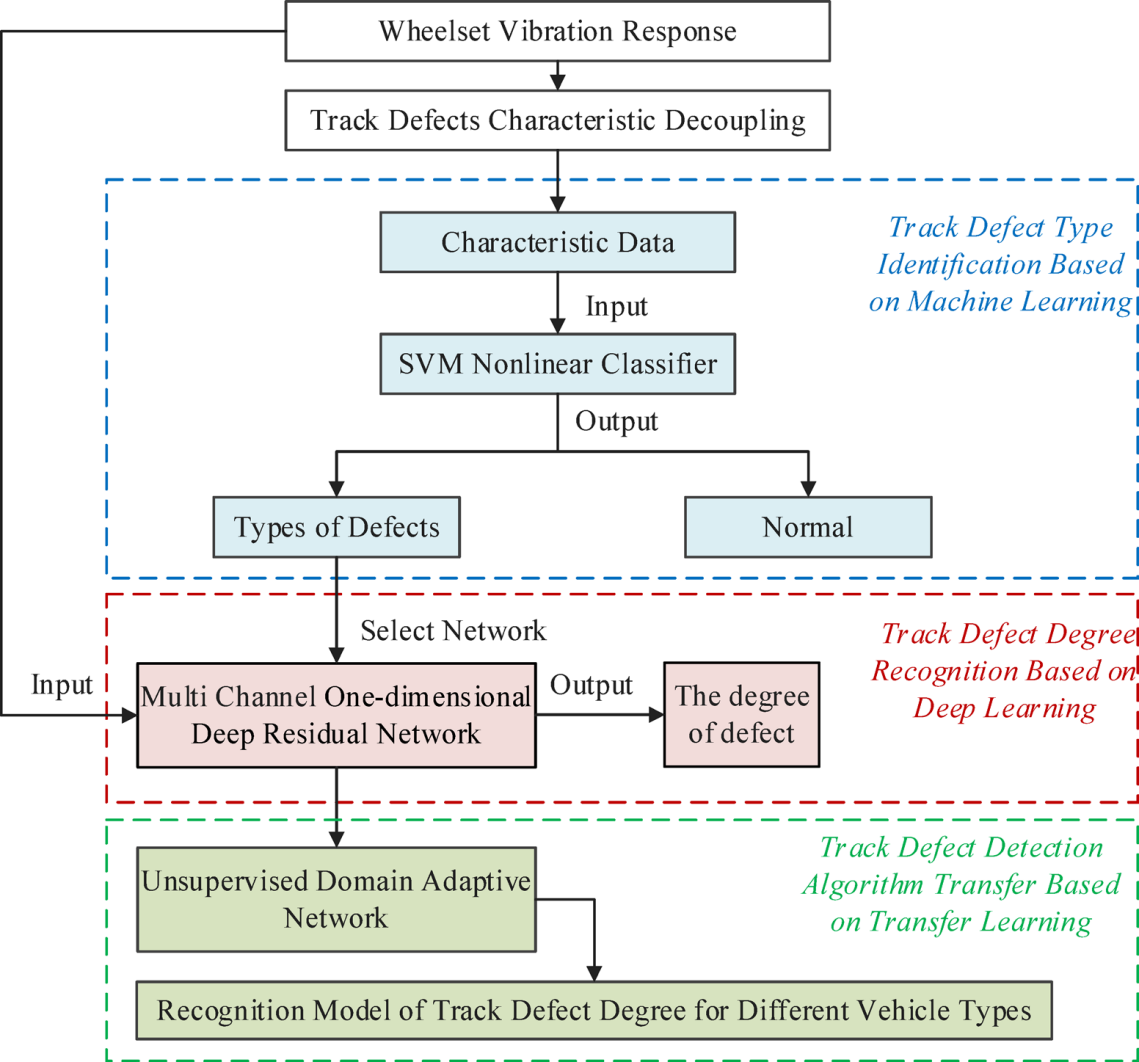
Flow chart of multi-stage intelligent detection algorithm of track defects.

After obtaining the vibration response feature data of wheelsets under track defects, it is necessary to locate, classify, and estimate the degree of the track defects. Based on the decoupling algorithm of track defect features, a three-stage network model combining traditional machine learning, deep learning, and transfer learning is constructed to study intelligent algorithms for identifying track defect types and estimating defect degrees. A multi-stage intelligent detection algorithm of track defects is proposed, as shown in Fig. 11.

In the first stage, a track defect type identification algorithm is developed based on traditional machine learning theory. The Support Vector Machine (SVM) nonlinear classifier is employed to quickly classify the input feature data of wheelset vibration response, enabling fast identification of track defects, including rail corrugation depth levels, fastener looseness, and CA mortar disengagement.

In the second stage, a precise recognition model for track defect degree is constructed based on deep learning theory. A one-dimensional deep residual network is used to input multi-channel vibration acceleration data from multiple wheelsets, and separate training is performed for different track defects to achieve accurate recognition of defect degree for different track defects, including rail corrugation depth, number of fastener looseness, and CA mortar disengagement length.

In the third stage, an unsupervised domain adaptive track defect recognition transfer model is developed based on transfer learning theory. A multiple feature moment matching method is employed to adapt the track defect degree recognition model to different vehicle types, enhancing the generalization of the track defect degree recognition algorithm.

### Track defect type identification based on track defect characteristics

**Fig. 12:**
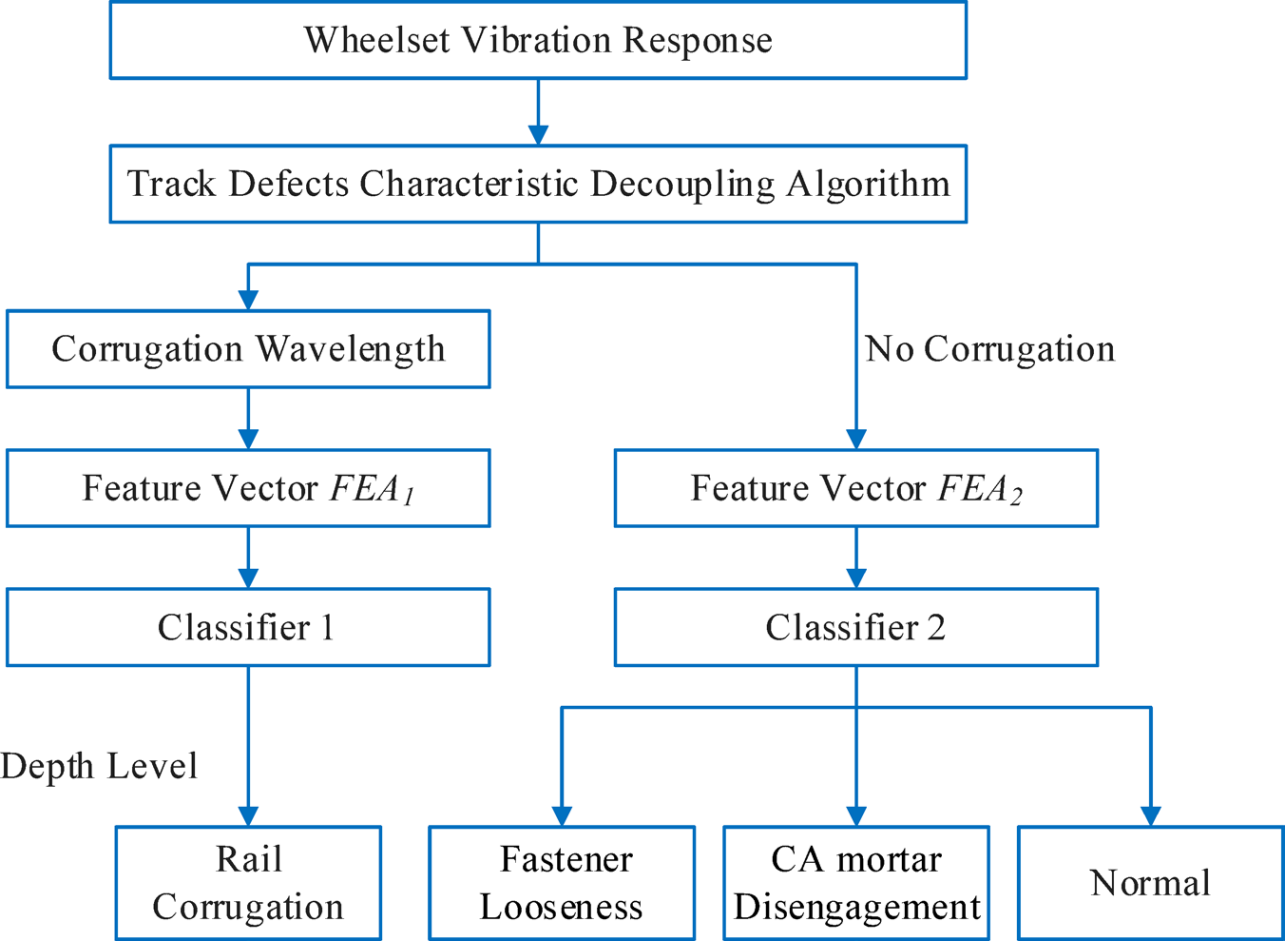
Flow chart of track defect type identification based on track defect characteristics.

The traditional machine learning model offers the advantages of a lightweight network structure and quick training speed. Therefore, a track defect type identification algorithm based on the SVM nonlinear classifier is proposed to locate and identify track defects efficiently. A flow chart for identifying track defect types based on defect characteristics is shown in Fig. 12. Using two nonlinear classifier models to quickly categorize the characteristic data of wheelset vibration responses, identify typical defects such as rail corrugation, fastener loosening, and CA mortar layer disengagement, and combine sliding window analysis of signals to locate defects along track mileage.

Construct a wheelset vibration response feature vector ***FEA***_***1***_ to serve as the input for Classifier 1 in the track defect type identification algorithm. This vector is used for identifying and grading rail corrugation. The feature vector ***FEA***_***1***_ includes vehicle speed, an estimated value of the rail corrugation wavelength, and high-order spectral features of the wheelset vertical vibration response, expressed as

20 $$FE{A_1}=\left[ {V,\lambda ,{H_1},{H_2},{H_3},{H_4},{H_5},{H_6},{H_7},{H_8},{H_9}} \right]$$

Construct a wheelset vibration response feature vector ***FEA***_***2***_ to serve as input for Classifier 2 in the track defect type identification algorithm, aimed at identifying fastener looseness and CA mortar layer disengagement. The wheelset vibration response feature vector FEA2 includes vehicle speed, PSD variation index, and time-frequency feature values of wheelset vertical vibration response, expressed as

21 $$FE{A_2}=\left[ {V,E{I_V},T_{1}^{V},T_{2}^{V}, \ldots ,T_{{12}}^{V},E{I_L},T_{1}^{L},T_{2}^{L}, \ldots ,T_{{12}}^{L}} \right]$$

Divide the rail corrugation into three levels based on the depth of the corrugation. The rail corrugation with a depth of 0.04 mm to 0.05 mm is classified as Level 1, the rail corrugation with a depth of 0.06 mm to 0.08 mm is classified as Level 2, and the rail corrugation with a depth of 0.09 mm to 0.1 mm is classified as Level 3. The classifiers adopt the Naive Bayes Model (NBM), the Radial Basis Function (RBF) network, and the SVM, respectively.

Randomly select 80% of the data for training and the remaining 20% for testing, while ensuring each class is balanced. The identification accuracy of track defects is defined as the ratio of the number of correctly classified track defect types in the test set to the total number of test set. The results of track defect identification are shown in Table 8. The accuracy of the SVM nonlinear model for corrugation depth classification exceeds 97%, and its accuracy in identifying fastener looseness and CA mortar disengagement is over 84%. Moreover, the differences in results between different cases are slight, indicating that using an SVM nonlinear model as a classifier has stronger generalization.

**Table 8:** Identification accuracy of track defects.

Classifier		Case 1	Case2	Case 3	Case 4	Average
Classifier 1	NBM	52.38%	55.36%	59.23%	61.01%	57.00%
	RBF	80.65%	66.07%	83.04%	51.49%	70.31%
	SVM	99.40%	98.51%	100%	97.32%	98.66%
Classifier 2	NBM	87.30%	86.51%	85.32%	86.11%	86.31%
	RBF	53.57%	11.11%	15.87%	10.32%	22.72%
	SVM	92.86%	86.11%	92.86%	84.52%	89.09%

To verify the robustness of the track defect characteristic decoupling algorithm and track defect type identification, different levels of white noise are added to the signal to simulate measurement noise. The identification results under various noise conditions are shown in Table 9. The identification accuracy across various noise levels remains fairly stable, showing that the proposed algorithm for identifying track defect types is less impacted by signal noise.

**Table 9:** Identification accuracy of track defects under different noises.

Classifier	Noise	Case 1	Case2	Case 3	Case 4	Average
Classifier 1	1%	99.40%	98.51%	100%	96.73%	98.66%
	3%	99.40%	99.40%	99.70%	97.02%	98.88%
	5%	99.40%	98.21%	100%	96.43%	98.51%
Classifier 2	1%	94.44%	88.89%	92.06%	86.90%	90.57%
	3%	90.87%	86.11%	86.90%	86.11%	87.50%
	5%	89.29%	82.94%	85.71%	84.92%	85.72%

Conventional signal decomposition algorithms such as Empirical Mode Decomposition (EMD), Variational Mode Decomposition (VMD), and WPD are used to break down the wheelset vibration acceleration signal, and the energy values of each component are extracted as feature parameters for identifying track defects. The parameters of all compared decomposition algorithms are the same as those in the proposed track defects characteristic decoupling algorithm. Table 10 compares the accuracy of track defect identification using the characteristic decoupling algorithm proposed in this paper and other feature extraction methods, which validates the effectiveness of the wheelset vibration response feature vectors *FEA*_*1*_ and *FEA*_*2*_ constructed based on the track defect characteristic decoupling algorithm.

**Table 10:** Identification accuracy of track defects under different feature extraction methods.

Decoupling Algorithm	Features	Identification Accuracy of Classifier 1	Identification Accuracy of Classifier 2
EMD	The Energy Values of Each Component	74.40%	66.67%
VMD	The Energy Values of Each Component	77.38%	70.83%
WPD	The Energy Values of Each Component	83.33%	75.00%
Proposed	*FEA*_*1*_ and *FEA*_*2*_	98.66%	89.09%

### Track defect degree recognition based on a one-dimensional deep residual network

Because of the nonlinear response of wheelset vibration caused by the combined effects of multiple factors, shallow network models struggle to accurately describe the complex relationship between wheelset vibration response signals and track defect severity. Deep learning, with its deep nonlinear network structure and integrated feature learning, can automatically learn features and has a stronger ability to represent complex wheelset vibration signals. After quickly identifying track defects, a precise end-to-end model for estimating the degree of track defects is designed using one-dimensional convolution and the residual network. The network takes multi-channel vibration response acceleration signals from multiple wheelsets as input, and its output is rail corrugation depth, the number of fastener looseness, or CA mortar disengagement length. The recognition models for rail corrugation depth, number of fastener looseness, and CA mortar disengagement length are all built with the same network structure and trained independently. According to the classification of the track defect type identification algorithm, select the corresponding degree recognition model based on the track type to recognize the degree of track defects accurately. Using integrated multiple network models makes it easier to identify track defects and improves the accuracy of estimating the severity of defects compared to using a single network for multiple recognition tasks.

The vibration response data of a wheelset consists of 5 channels, i.e., a data matrix with 5 rows and *N* columns, expressed as

22 $$Dat{a_i}=\left[ {\begin{array}{*{20}{l}} {{\boldsymbol{\lambda}_i}} \\ {\hat {{\boldsymbol{A}}}_{i}^{V}} \\ {\hat {{\boldsymbol{A}}}_{i}^{L}} \\ {{\boldsymbol{A}}_{i}^{V}} \\ {{\boldsymbol{A}}_{i}^{L}} \end{array}} \right]$$

where, $\lambda$ is the estimated wavelength of rail corrugation, which is zero if there is no corrugation; ${\hat {A}^V}$ and ${\hat {A}^L}$ are the vertical and lateral vibration accelerations of the wheelset under normal conditions, respectively; ${A^V}$ and ${A^L}$ are the vertical and lateral vibration accelerations of the wheelset to be recognized.

In addition to the vibration acceleration that needs to be recognized‌, the vibration response data of a wheelset also includes the acceleration signal of the wheelset vibration response under normal conditions, so the input data of the recognition network contains information about track roughness. The sampling frequency remains constant, but when trains pass through the same length of track at different speeds, the length of vibration acceleration data becomes inconsistent. To facilitate model input, zero padding is used at the end of the signal to standardize the length of vibration acceleration signals at different vehicle speeds, ensure data alignment, and include vehicle speed information in the wheelset vibration response data. Under the premise of balancing various working conditions, 80% of the wheelset vibration response data is randomly selected as the training set for the track defect degree recognition network, and the remaining 20% is used as the testing set for the track defect degree recognition network.

**Fig. 13:**
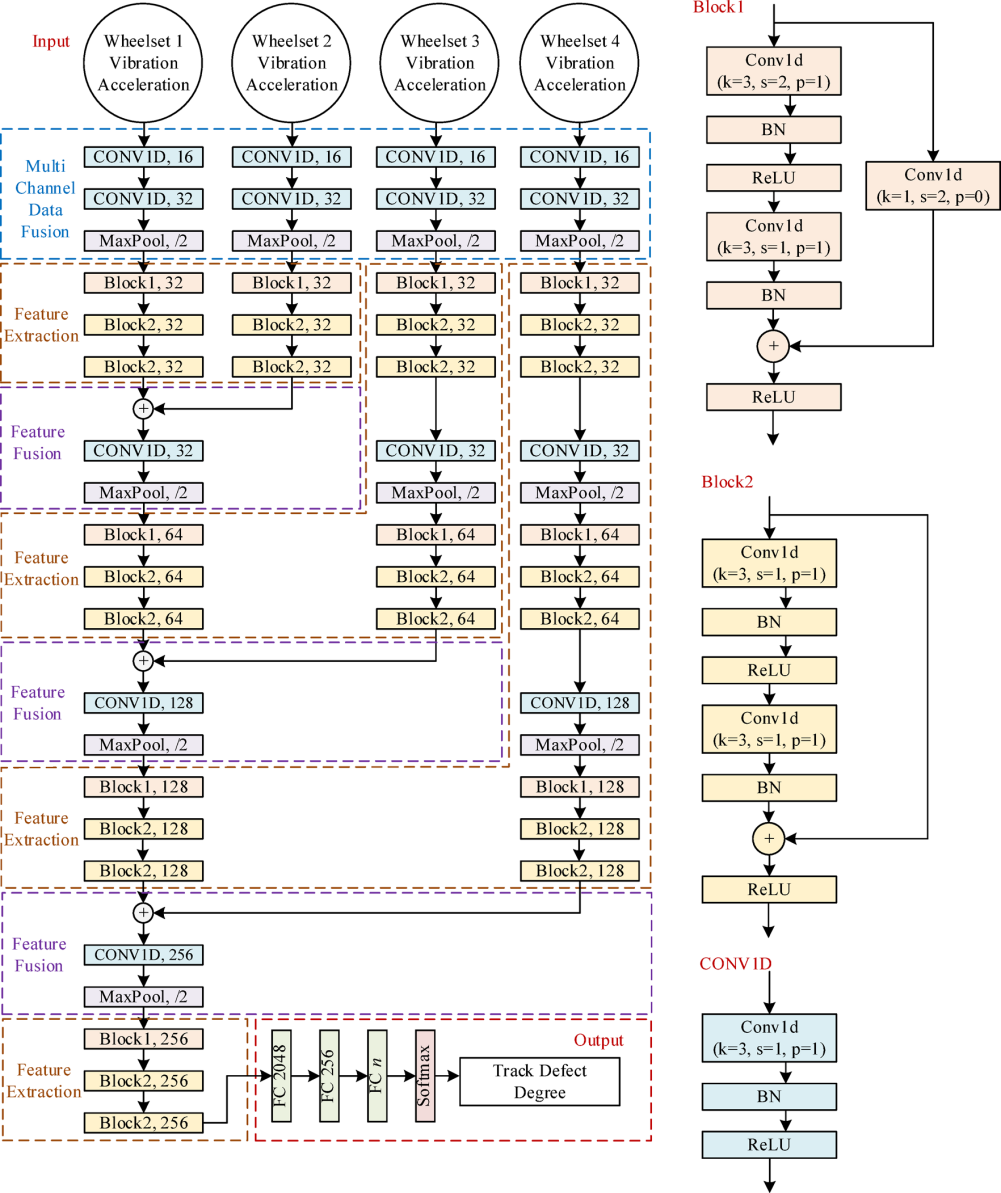
Track defect degree recognition based on a one-dimensional deep residual network.

Design a multi-channel model for accurate recognition of track defect degree based on a one-dimensional convolutional residual network structure and multiple wheelset data. The network structure of the track defect degree recognition model is shown in Fig. 13.

The input of the precise recognition model for track defect degree is the vibration response of four wheelsets, and the vibration response data of each wheelset is composed of one-dimensional data from five channels. The recognition model for track defect degree first performs data fusion on each wheelset, then extracts features. Based on the actual situation of four wheelsets passing through a track with defects in sequence, the accurate track defect degree recognition model combines data features from different wheelsets, adopting an alternating approach of feature fusion and feature extraction to integrate the vibration response characteristics of the four wheelsets gradually. The feature fusion module consists of one-dimensional convolutional network feature fusion blocks and pooling layers, while the feature extraction module consists of multiple one-dimensional residual convolution blocks. The output layer of the model is equipped with three fully connected layers and a SoftMax function.

The track defect degree recognition accuracy is defined as the ratio of the number of correctly recognized track defect degrees in the test set to the total number of test set. The recognition results of rail corrugation depth, number of fastener looseness, and CA mortar disengagement length are shown in Table 11. The deep learning-based network model can effectively learn the nonlinear relationship between wheelset vibration responses and the degree of track defects. The combination of vibration responses from multiple wheelsets will further improve the accuracy of the model in recognizing‌ the degree of track defects. The designed model applies to three different types of track defects, eliminating the need to create separate network structures for each, which demonstrates the versatility of the proposed algorithm.

**Table 11:** Track defect degree recognition accuracy.

Track Defect	Input Data	Model	CRH3	CRH2
Rail Corrugation	Feature Vector	SVM	96.57%	94.94%
	1 Wheelset Data	Proposed	99.10%	99.94%
	4 Wheelsets Data	Proposed	**100%**	**100%**
Fastener Looseness	Feature Vector	SVM	60.92%	44.64%
	1 Wheelset Data	Proposed	91.22%	89.44%
	4 Wheelsets Data	Proposed	**99.67%**	**96.33%**
CA Mortar Layer Disengagement	Feature Vector	SVM	47.35%	40.90%
	1 Wheelset Data	Proposed	90.08%	91.16%
	4 Wheelsets Data	Proposed	**99.58%**	**99.67%**

**Fig. 14:**
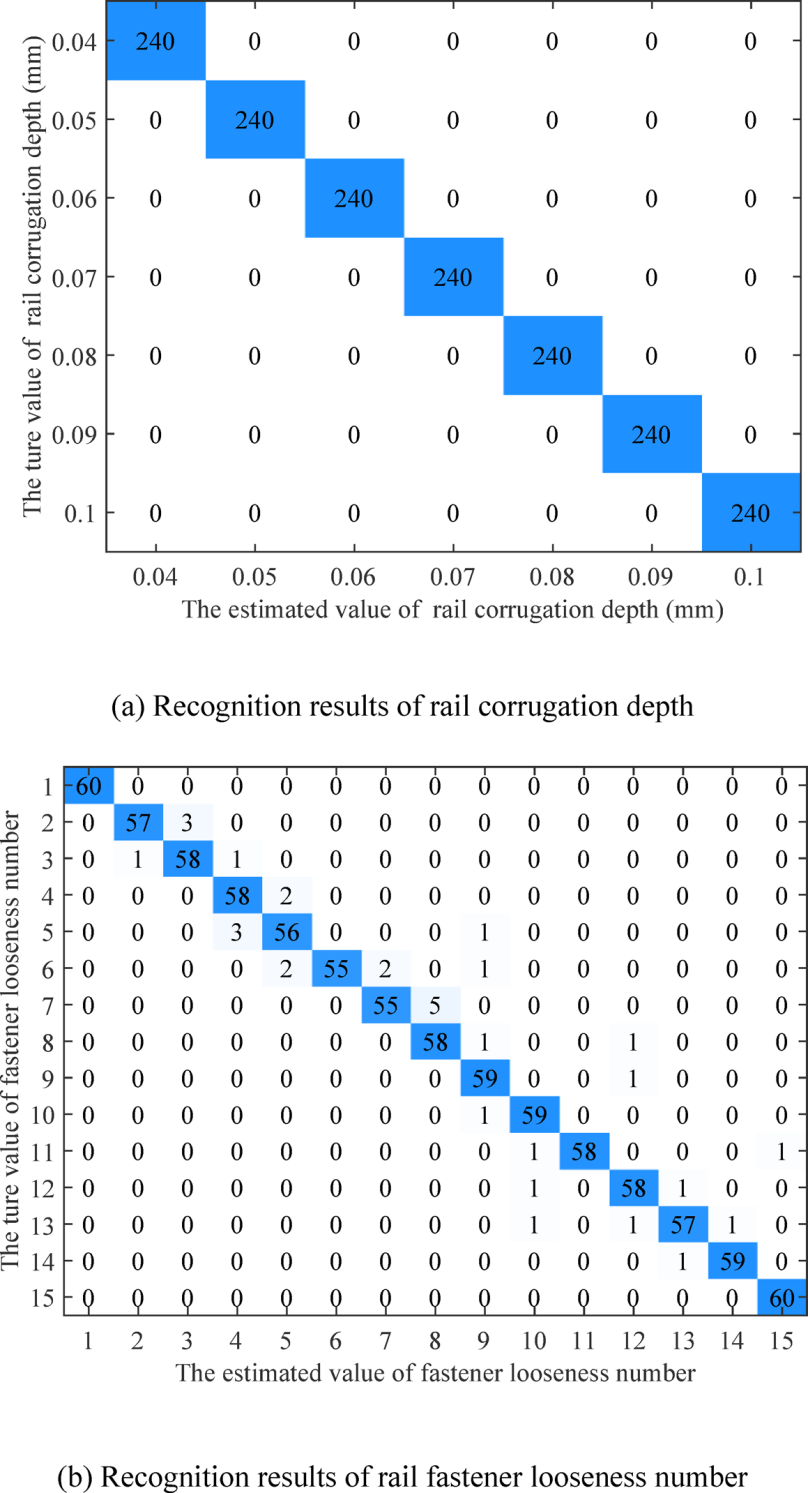

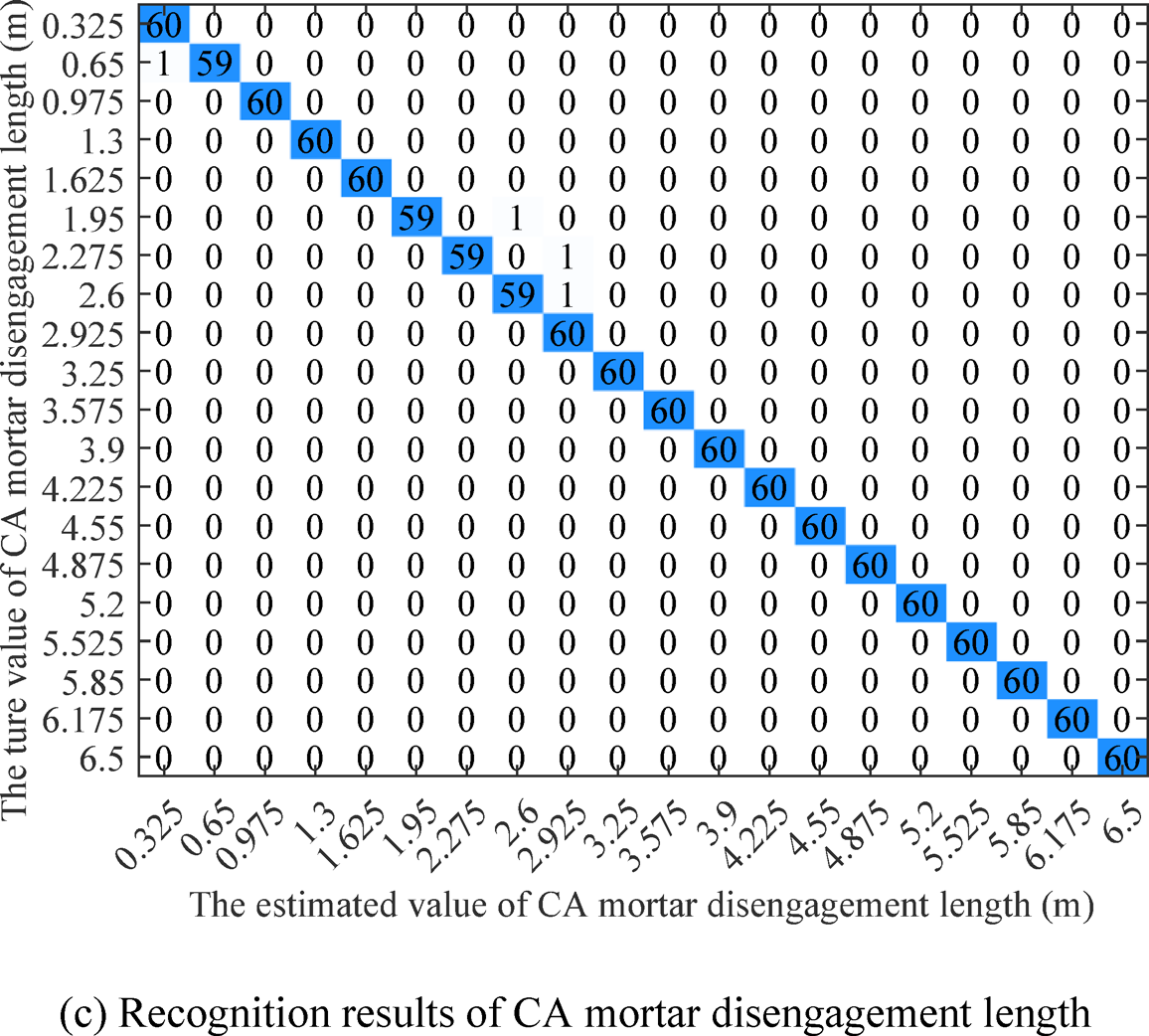
The confusion matrix of Track defect degree recognition.

The confusion matrices of track defect degree recognition are shown in Fig. 14. The analysis in Sect. 3.1 demonstrates that rail corrugation significantly affects the wheelset vibration response at high vehicle speeds. Therefore, when using traditional feature extraction or single wheelset data for rail corrugation depth recognition, the accuracy remains very high. The confusion matrix shows that the proposed algorithm can recognize all rail corrugation depths. The macro-precision of fastener looseness number recognition is 96.40%, the micro-precision is 96.33%, the macro-recall is 96.33%, and the micro-recall is 96.33%, all of which are close to the overall accuracy. The macro-precision, the micro-precision, the macro-recall, and the micro-recall of CA mortar disengagement length recognition are all 99.67%, all of which are also close to the overall accuracy. The incorrect recognition values of the fastener looseness number and CA mortar disengagement length are both around the true values, which aligns with the characteristic that it is hard to distinguish minor defects with similar degrees, and it also confirms the analysis in Sect. 3.2 and 3.3. In addition, the data in the test set is not used for training, and the accuracy of the test set is similar to the validation accuracy of the training set.

To verify the robustness of the algorithm, different sampling frequencies are used for the wheelset vibration acceleration signal. The accuracy of track defect degree recognition at various sampling frequencies is shown in Table 12. Under different sampling frequency conditions, the average accuracy of recognizing the depth of rail corrugation, the number of fastener looseness, and the length of CA mortar disengagement surpasses 96%, indicating that the sampling frequency of the signal has a relatively small impact on track defect degree recognition. Therefore, a relatively low sampling frequency can be used to decrease the amount of data input to the model, enhance the training speed, and improve the detection efficiency of the model.

General Convolutional Neural Network (CNN), Residual Network (ResNet), Recurrent Neural Network (RNN), and Long Short-Term Memory (LSTM) are used to recognize the degrees of track defects, and the identification results are presented in.

Table 13. The number of network layers and output layer structures in all comparison models are the same as those in the proposed track defect degree recognition model. The proposed track defect degree recognition model performs best in recognizing the depth of rail corrugation, the number of fastener looseness, and the length of CA mortar disengagement, demonstrating the effectiveness of the algorithm.

**Table 12:** Track defect degree recognition accuracy under different sampling frequencies.

Track Defect	Sampling Frequency	CHR3	CRH2	Average
Rail Corrugation	10k	100.00%	100.00%	100.00%
	5k	100.00%	100.00%	100.00%
	2.5k	99.94%	99.76%	99.85%
Fastener Looseness	10k	99.67%	96.33%	98.00%
	5k	99.78%	96.44%	98.11%
	2.5k	99.56%	96.56%	98.06%
CA Mortar Layer Disengagement	10k	99.58%	99.67%	99.63%
	5k	99.41%	99.58%	99.50%
	2.5k	99.67%	99.75%	99.71%

**Table 13:** Track defect degree recognition accuracy under different recognition models.

Track Defect	Recognition Model	Recognition Accuracy
Rail Corrugation	CNN	96.61%
	ResNet	97.98%
	RNN	89.29%
	LSTM	92.26%
	Proposed	**99.94%**
Fastener Looseness	CNN	67.71%
	ResNet	77.78%
	RNN	58.33%
	LSTM	64.58%
	Proposed	**99.56%**
CA Mortar Layer Disengagement	CNN	61.42%
	ResNet	72.50%
	RNN	57.50%
	LSTM	62.5%
	Proposed	**99.67%**

### Track defect detection algorithm transfer based on multiple feature moment matching

In order to improve the generalization of the track defect recognition algorithm across different vehicles, the model is adapted to various vehicle types using transfer learning. By employing a domain adaptive approach, the deep network trained on one vehicle can be applied to a new vehicle, allowing cross-device model transfer without relying on prior knowledge or large amounts of labelled data. This increases the flexibility of track defect recognition algorithms.

**Fig. 15:**
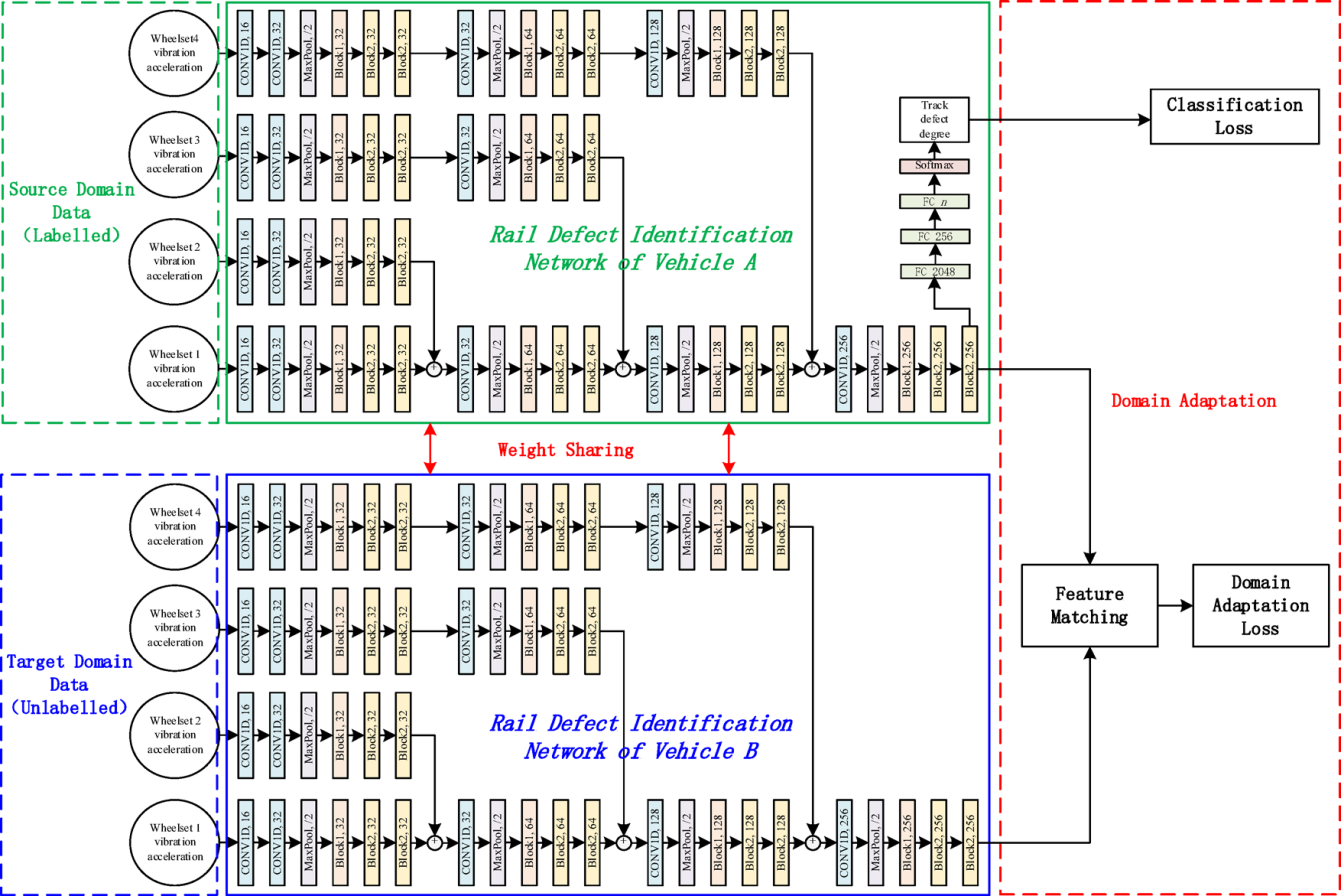
Track defect detection algorithm transfer network based on the depth transfer learning for different vehicle types.

The main goal of unsupervised domain adaptation is to align features across different domains and minimize the disparities in their feature distributions. A deep transfer learning network for recognizing the track defect degree across different vehicle types was designed based on the unsupervised domain adaptive method, which is shown in Fig. 15. The wheelset vibration response data of vehicle A is labelled, and this data is referred to as the source domain data. The wheelset vibration response data of vehicle B is unlabelled, and this data is referred to as the target domain data. By reducing the difference in feature distribution between the source and target domain datasets, the transfer of the track defect detection algorithm between vehicle A and vehicle B can be achieved. Sharing network parameters, such as network weights, for track defect degree recognition models across different vehicles. In the domain adaptation layer, feature matching methods are used to compare the source and target domain features and calculate the loss between them.

The maximum mean difference (MMD) is defined as the difference between the means of two domain feature distributions mapped into a high-dimensional space, expressed as

23 $$MMD\left( {{F_S},{F_T}} \right)={\left\| {\frac{1}{{{n_S}}}\sum\limits_{{i=1}}^{{{n_S}}} {\phi \left( {{F_{Si}}} \right)} - \frac{1}{{{n_T}}}\sum\limits_{{j=1}}^{{{n_T}}} {\phi \left( {{F_{Tj}}} \right)} } \right\|^2}$$

where, ${F_S}$ is the feature of the source domain data, which is the output vector of the source domain data in the domain adaptation layer; ${n_S}$ is the number of samples in the source domain data; ${F_T}$ is the feature of the target domain data, which is the output vector of the target domain data in the domain adaptation layer; ${n_T}$ is the number of target domain data samples; $\phi (.)$ represents a nonlinear mapping function, usually using a Gaussian function.

The second-order feature transformation CORAL method computes the covariance difference between two domain feature distributions, expressed as

24 $$CORAL\left( {{F_S},{F_T}} \right)=\frac{1}{{4{d^2}}}{\left\| {C\left( {{F_S},} \right) - C\left( {{F_T}} \right)} \right\|^2}$$

where, *d* is the size of the eigenvalue dimension; $C(.)$ represents the covariance matrix.

Based on Euclidean distance, define the multi-order moment (MOM) of two domain feature distributions, expressed as

25 $$\begin{gathered} MM\left( {{F_S},{F_T}} \right)=\left\| {E\left( {{F_S}} \right) - E\left( {{F_T}} \right)} \right\| \\ +\sum\limits_{{m=1}}^{M} {\left\| {E\left[ {{{\left( {{F_S} - E\left( {{F_S}} \right)} \right)}^m}} \right] - E\left[ {{{\left( {{F_T} - E\left( {{F_T}} \right)} \right)}^m}} \right]} \right\|} \\ \end{gathered}$$

where, $E(.)$ represents expectation.

MMD describes the overall similarity of different feature distributions. CORAL reflects the statistical characteristics of two different datasets. MOD clarifies the nonlinear relationship between various data distributions. A multiple feature moment matching (MFMM) method based on MMD, CORAL, and MOM is proposed to measure differences in domain features from numerous perspectives, including edge distribution features, statistical features, and nonlinear features. The objective function of the transfer learning network is

26 $$\hbox{min} \frac{1}{N}\sum\limits_{{i=1}}^{N} {J\left( {\theta ({x_i}),{y_i}} \right)} +{k_1}\cdot MMD\left( {{F_S},{F_T}} \right)+{k_2}\cdot CORAL\left( {{F_S},{F_T}} \right)+{k_3}\cdot MM\left( {{F_S},{F_T}} \right)$$

where, $J(.)$ represents the cross entropy loss between the network output and the label; $\theta (.)$ represents the weight parameters for network optimization; *k*_*1*_、*k*_*2*_ and *k*_*3*_ are the domain adaptation coefficients, respectively.

The transfer results of the degree recognition algorithm for rail corrugation depth, number of fastener looseness, and CA mortar disengagement length under different domain adaptive methods are shown in Table 14. The results indicate that the transfer learning model using the multiple feature moment matching method has better transfer performance than the transfer learning model using a single feature moment matching method, and is suitable for different transfer tasks of different track defect degree recognition algorithms.

**Table 14:** Transfer results of track defect degree recognition network.

Track Defect	Domain Adaptive Method	CHR3 → CRH2	CRH2 → CRH3	Average
Rail Corrugation	Not transfer	56.73%	41.07%	48.90%
	MMD	78.09%	70.30%	74.20%
	CORAL	71.07%	61.96%	66.52%
	MOM	72.26%	62.02%	67.14%
	MFMM	**79.35%**	**76.79%**	**78.07%**
Fastener Looseness	Not transfer	56.44%	68.88%	62.66%
	MMD	92.22%	93.33%	92.78%
	CORAL	88.89%	91.56%	90.23%
	MOM	78.22%	87.22%	82.72%
	MFMM	**97.56%**	**97.00%**	**97.28%**
CA Mortar Layer Disengagement	Not transfer	76.25%	78.33%	77.29%
	MMD	96.83%	97.92%	97.38%
	CORAL	95.25%	96.58%	95.92%
	MOM	95.83%	96.75%	96.29%
	MFMM	**99.58%**	**99.67%**	**99.63%**

## Conclusion

The paper centers on an intelligent recognition algorithm for high-speed railway track defects based on the wheelset dynamic response of the vehicle. The main research work of the paper are as follows:

(1) The established multi-scale track defect model and the proposed segmental numerical solution method allow the vehicle-track simulation model to simulate various degrees of rail corrugation, fastener looseness, and CA mortar disengagement. They also enhance simulation efficiency and provide a data foundation for researching on-board detection algorithms for track defects.

(2) Based on analyzing the impact of track defects on the vibration response of wheelsets, a nonlinear track defect feature decoupling algorithm is proposed that uses signal decomposition and time-frequency analysis. By examining the frequency of characteristic components, the wavelength of rail corrugation can be estimated, and the constructed *DCI* increases with the depth of the corrugation. Additionally, the created *VDI* and *LDI* can differentiate between fastener looseness and CA mortar disengagement. The results demonstrate that the developed defect feature index can effectively capture the vibration response characteristics resulting from track defects.

(3) A multi-stage intelligent detection algorithm for track defects based on non-linear classifiers, a multi-channel deep residual network, and multiple feature moment matching has been put forward to improve the accuracy and generalization of track defect recognition. The proposed algorithm enables the identification of track defect types, the accurate recognition of defect degree, and the transfer of the algorithm across different vehicle types.

The algorithm research discussed in this article is based on simulation data, providing a theoretical basis for engineering applications. In the future, the plan is to collect vehicle vibration response signals from real trains and further research the on-board detection technology for track defects.

## Data Availability

All data generated or analysed during this study are included in this published article.
